# Rapidly increasing cyanobacteria blooms in the subarctic Great Slave Lake: observations from Indigenous, local, and scientific knowledge

**DOI:** 10.1038/s41598-025-07432-5

**Published:** 2025-07-08

**Authors:** Jeffrey Cederwall, Peter A. Cott

**Affiliations:** 1 Department of Environment and Climate Change, Government of the Northwest Territories, 5102 – 50th Ave., Yellowknife, NT X1A 2L9 Canada; 2 Present Address: Canada Water Agency, 5019 – 52nd St., Yellowknife, NT X1A 1T5 Canada

**Keywords:** Indigenous Knowledge, Climate change, Cumulative impacts, Northwest Territories, Phytoplankton, Oligotrophic, Limnology, Climate-change ecology, Freshwater ecology

## Abstract

Cyanobacteria blooms, typically associated with warm, eutrophic lakes, are increasingly observed in colder, oligotrophic lakes. Cyanobacteria blooms can have ecological impacts and pose health risks when composed of toxin-producing taxa. By combining Indigenous, local, and scientific knowledge sources, we document a profound shift in Great Slave Lake—a huge, remote, oligotrophic lake in Northwest Territories, Canada. Suspected blooms were first observed in 1989, localized near point-source sewage effluent. Since 2009, sporadic blooms have appeared in new areas of the North Arm, away from known point source nutrients, and become increasingly frequent. By 2020, bloom density increased, with the densest and most widespread blooms observed in 2024. These blooms have generally been nearshore and transient, most frequently located in sheltered waters, which are warmer and shallower relative to the rest of the lake. *Dolichospermum* is the dominant genus, with no microcystin toxins detected. We hypothesize these unprecedented blooms may be climate-driven, enabled by a combination of warmer water, reduced wind and ice cover, and potentially fueled by nutrient inputs from record water levels, intensified wildfires, permafrost thaw, and cultural eutrophication. By synthesizing across knowledge systems, we establish a foundation for collaborative research and monitoring in rapidly changing northern water bodies.

**Supplementary Information:**

The online version contains supplementary material available at 10.1038/s41598-025-07432-5.

## Introduction

Climate change is driving rapid and diverse environmental changes across the Arctic and subarctic regions^1,2^. Northern regions are warming four times faster than the global rate^3^ and experiencing reduced ice cover^4^ and increased weather variability^5^. These rapid environmental changes can have profound and cascading ecological effects^6^, fueling concerns of crossing ecological tipping points^7^. Lakes are sentinels of environmental change and reflect the direct physical effects of climate change on arctic and subarctic freshwater lakes^8^. These changes have caused biological regime shifts in lakes^9^, such as the intensification of algal blooms^10^. Climate warming is causing northern lakes to stratify sooner and longer^11^, resulting in a longer open-water season that increases productivity^12^. These climate-driven effects have now reached the lower aquatic food webs of even very deep northern lakes, including Great Slave Lake, Northwest Territories (NWT), Canada^4^.

The emergence and intensification of surface algal blooms are readily observable indicators of water quality. They often indicate nutrient-rich, stagnant waters, and can potentially produce toxins which are harmful to wildlife and people. Across the globe, jurisdictions have adopted various quantitative definitions of what an algal bloom is, based on thresholds of biomass or the presence of toxins^13^. To align with Indigenous and local knowledge, we use the qualitative definition of algal blooms in this article. An algal bloom is recognized by the rapid growth of algae and/or cyanobacteria (also referred to as blue-green algae) through a visible change in the water’s clarity or colour, often with a surface scum^14^. Conventionally, algal blooms are considered problems of nutrient enrichment and warm water^15,16^, and therefore not typically associated with cold, nutrient-poor northern waters that are commonly covered in ice for greater than six months of the year. Well-established research has demonstrated nutrients, particularly phosphorus, fuel algal blooms^17,18^ with new research continuing to support nutrient inputs, not temperature, as the primary driver^19^. However, under a rapidly changing climate, a growing number of temperate lakes are experiencing blooms, including some cold, oligotrophic lakes^20^. Even so, the deep subarctic waters of Great Slave Lake have long been considered too cold and unproductive to support blooms^21^.

Bridging and co-production of Indigenous Knowledge and science is advancing our collective understanding of the environmental and ecological effects of climate change, particularly in the Arctic and subarctic^22^. Indigenous Knowledge (also referred to as Traditional Knowledge in the NWT and elsewhere) is knowledge and values acquired through experience, observation, and from the land or spiritual teachings; handed down through generations^23^ and considers the environment holistically. Local knowledge is also place-based knowledge, gained through experience and observation of the land but is not necessarily generational and may be held by both Indigenous and non-Indigenous peoples. When Indigenous and local knowledge systems are respectfully bridged with science to co-produce research, we can gain greater insight into ecological changes and can extend the temporal scale of our understanding^24^. This knowledge bridging may be particularly important in the north, where scientific research and monitoring has been greatly limited by the remoteness of these regions.

Despite Great Slave Lake’s ecological, cultural, and economic importance, scientific research and monitoring have been extremely limited relative to the Laurentian Great Lakes and other large lakes worldwide^25^. Today, the most comprehensive scientific assessment of Great Slave Lake remains the lake-wide assessment in 1944–1947^26,27^. Since then, most scientific research has been focused on fisheries^28^ or contaminants^29,30^. Unlike southern jurisdictions, where cyanobacterial blooms are tracked and publicly reported by the provinces, blooms are an emerging issue in the NWT and there is no established surveillance program. Here, land user observations are particularly important. Throughout the Great Slave Lake basin, the local population is accustomed to pristine drinkable freshwater, often paying close attention to their lands and waters, as many prefer to, and commonly do, drink directly from lakes and rivers.

Without previous algal bloom monitoring and only limited, mostly unpublished, scientific data, Indigenous and local knowledge is the most useful source of historical information. People have resided around the lake—often for generations—and can provide invaluable insights and observations of ecosystem change over time. Here, we document the emergence and increasing observations of cyanobacteria blooms on Great Slave Lake, from a combination of Indigenous, local, and scientific knowledge sources. We compiled a retrospective observational record of suspected cyanobacterial bloom occurrences across Great Slave Lake over time by reviewing surveys and interviews with long-time land and water users and summarizing their observations of overall ecosystem changes. Through opportunistic scientific sampling, we taxonomically identified the cyanobacterial taxa and screened for microcystin toxins. This work highlights the value and importance of using different knowledge systems when tracking ecosystem change over time, and is the first article to document widespread and increasing cyanobacteria blooms within Great Slave Lake.

## Methods

### Great Slave Lake

**Fig. 1:**
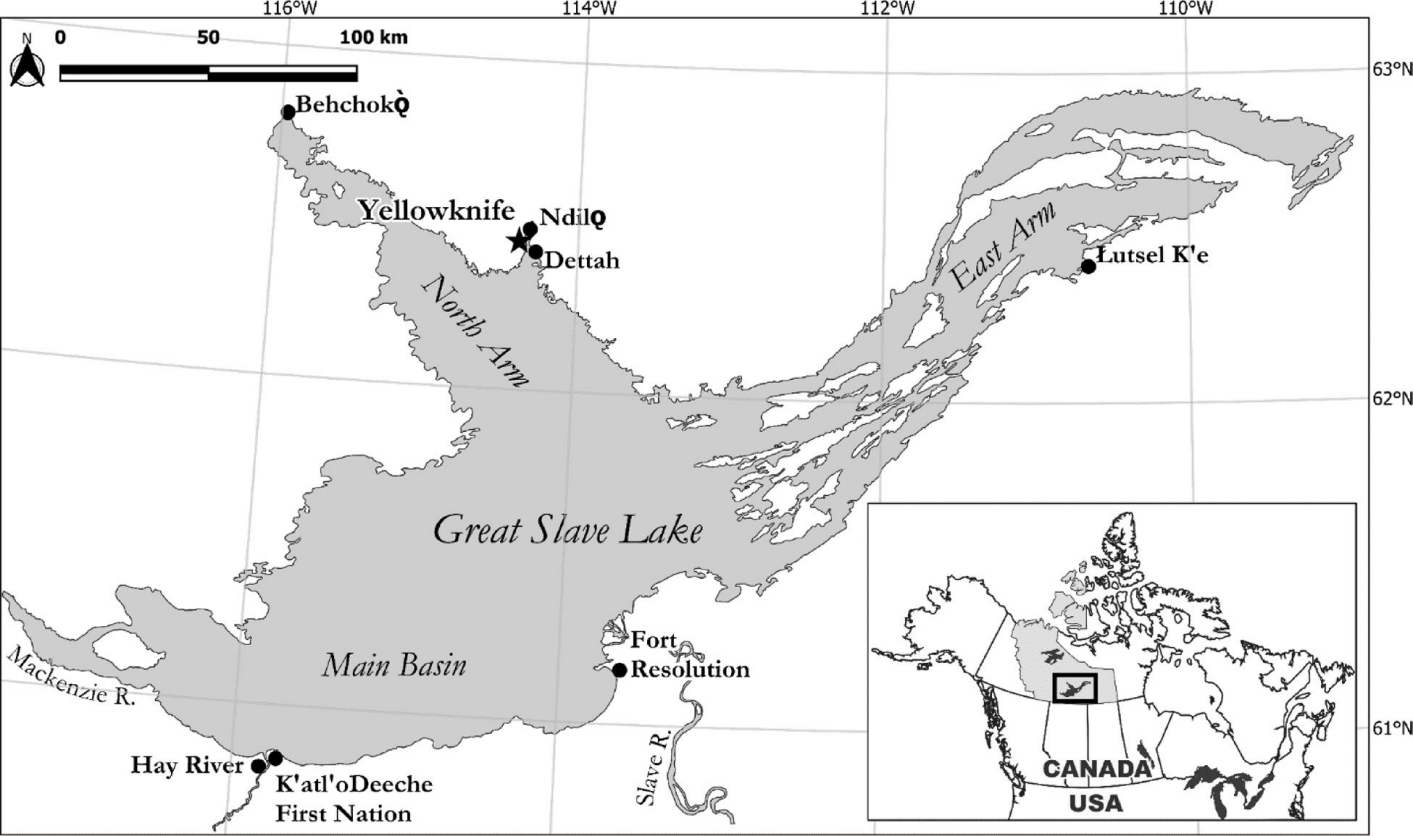
Great Slave Lake, Northwest Territories, Canada showing lake sections, major rivers, communities, and capital city. Note: Map created using QGIS 3.34.3 (https://www.qgis.org/).

Great Slave Lake is one of the world’s truly “great” freshwater lakes, being the 9th largest by area (28,568 km^2^), 9th by volume (2,088 km^3^), and the 4th deepest at 614 m^31^. This subarctic, oligotrophic lake is situated between 61° N and 63° N (Fig. 1), straddling the Taiga Plains ecozone to the west and the Taiga Shield ecozone to the north and east, a region characterized by long, cold winters and short warm summers and where ice cover typically lasts six months or more^32^. The lake has three sections: (1) the Main Basin, with a maximum depth of 163 m and containing the lake’s primary inflows (the Slave and Hay rivers) and outflow (the Mackenzie River), (2) the North Arm is shallower (max depth 106 m) with numerous nearshore islands and, (3) the East Arm, the deepest section of the lake (max depth 614 m), largely separated from the Main Basin by an extensive rocky archipelago. Surface waters are circulated through the Main Basin and North Arm via large counterclockwise gyres^33^. Based on the Organization for Economic Co-operation and Development (OECD) criteria for chlorophyll-a, Great Slave Lake is classified as overall oligotrophic and progresses to regionally ultra-oligotrophic further into the East Arm^27^. Total phosphorus is generally low in the northeast Taiga Shield waters; however, sediment plumes from the southern Slave River are unevenly deposited into the Main Basin, strongly influencing the lake. In 2023–2024, the median total phosphorus concentration was 7 µg/L (range: 2–13 µg/L) in the North Arm and 5 µg/L (range: 2–16 µg/L) in the East Arm (see Supplementary Table S1 and https://mackenziedatastream.ca/ for more data). Though there is no current Main Basin water chemistry, in 1993–1994, the Main Basin’s median total phosphorus during late winter was 17.5 µg/L (range: 9–26 µg/L)^34^, with the highest levels observed within the open-water sediment plume, reaching 47 µg/L (range: 13–227 µg/L)^30^. Great Slave Lake’s catchment basin spans 949,000 km^2^ and receives ~ 77% of its inflow from the turbid Slave River^35^. The Slave River annually delivers an enormous quantity of sediment (~ 6.7 × 10^10^ kg) containing substantial total phosphorus (~ 1.0 × 10^8^ kg), of which 80% is sediment-bound, into the Main Basin of Great Slave Lake^34,36^. This regular influx of nutrients has continued to fuel western and northern Canada’s largest freshwater commercial fishery of predominantly lake whitefish (*Coregonus clupeaformis*) and lake trout (*Salvelinus namaycush*)^37^.

Approximately 30,000 of NWT’s population of 45,000 reside adjacent to Great Slave Lake, including Yellowknife (the territorial capital), Hay River, and the largely Indigenous communities of Behchokǫ, Fort Resolution, Łutsël K’é, K‘atl’oDeeche First Nation, Ndilǫ, and Dettah^38^. The lake and its immediate headwaters provide drinking water for residents, both through community water treatment facilities and for those out on the land, where lake water is often consumed directly. The vast majority of Great Slave Lake’s extensive shoreline is undeveloped (Fig. 1). Local knowledge remains key for lake navigation as no comprehensive bathymetric maps exist.

### Surveys of Indigenous and local knowledge

As part of a collaborative Great Slave Lake research and monitoring program, observations on ecosystem-level changes over time from an Indigenous and local knowledge perspective were collected using surveys (see Supplementary Information 2a for participant disclosure). Surveys were administered within each Indigenous community through a local community coordinator to community-identified experts, who were compensated upon survey completion^39^. Participants were given the option to remain anonymous or provide their contact information for potential follow-up and updates. We subsequently conducted targeted, voluntary, semi-structured interviews with over 40 local experts (e.g., survey respondents, commercial fishermen, fishing lodge operators, environmental professionals, or those recommended by other land users). Questions included presence or absence, timing, appearance (water clarity, colour, texture, smell), location(s), phenology, severity of occurrence(s), and any other information they considered pertinent, such as photos (see Supplementary Information 2b for additional details). While the initial goal and focus of these surveys remained on Great Slave Lake, we also compiled additional observations of ecosystem change and suspected blooms from across the southern NWT.

We bridged Indigenous, local, and scientific knowledge through relationship building, mutual respect, and recognizing Indigenous and local knowledge as distinct knowledge sources, which we considered equal to science as credible evidence. Finally, we synthesized these Indigenous, local, and scientific knowledge sources to produce a timeline of ecological changes and a cyanobacteria occurrence map in Great Slave Lake.

### Scientific knowledge

To account for recorded observations from scientific or technical sources, we screened primary and grey literature, including scientific papers, environmental regulatory reports, technical regulatory or community workshop summaries, graduate theses, and media archives. We searched Google and Google Scholar for reports of suspected algal blooms using the keywords of “Great Slave Lake” coupled with “algae”, “algal, bloom”, “scum”, “cyanobacteria”, and “green stuff” (a colloquialism). To access this grey literature, we utilized the Mackenzie Land and Water Board’s public registry, the Government of the Northwest Territories departmental websites, the NWT Discovery Portal, and requested copies directly from authors or report holders.

In 2016, the Government of the Northwest Territories (GNWT) in partnership with Environment and Climate Change Canada, conducted aerial surveys on August 22, and September 3, 2016, to collect geotagged photographs of nearshore areas across the North Arm (See Supplementary Fig. S3 and S4 for flight path). GNWT manually classified each image for the visual presence or absence of both sediment and phytoplankton, then subjectively categorized the suspected phytoplankton into four abundance level categories: clear (none visible), low, medium, and high (see Supplementary Fig. S5–S8 for photographic examples). Only sites with highly abundant phytoplankton were considered suspected blooms. Starting in 2021, phytoplankton were opportunistically collected to confirm the taxonomy of suspected blooms; with samples taken between 0 and 30 cm beneath the surface, preserved with acid Lugol’s solution to 1% concentration and analyzed using a low-volume (1 mL) Utermöhl method and reported at the genus level (ALS Environmental Laboratory, Winnipeg, MB, see Supplementary Information 2d for more details). As of 2023, screening was included for total microcystin toxins, collected in amber glass bottles and stored in the fridge at 4 °C until analyzed by enzyme-linked immunosorbent assay (ELISA) with a 0.2 µg/L detection limit (DL; see Supplementary Information 2e for more details). Rapid toxin screening has been limited to microcystins due to their toxicity, frequent production, and potential persistence after blooms subside. In Canada, microcystins are the only class of cyanotoxins with established criteria: a maximum acceptable concentration of 1.5 µg/L for drinking water and a recreational water guideline of 10 µg/L^40,41^.

## Results and discussion

**Fig. 2:**
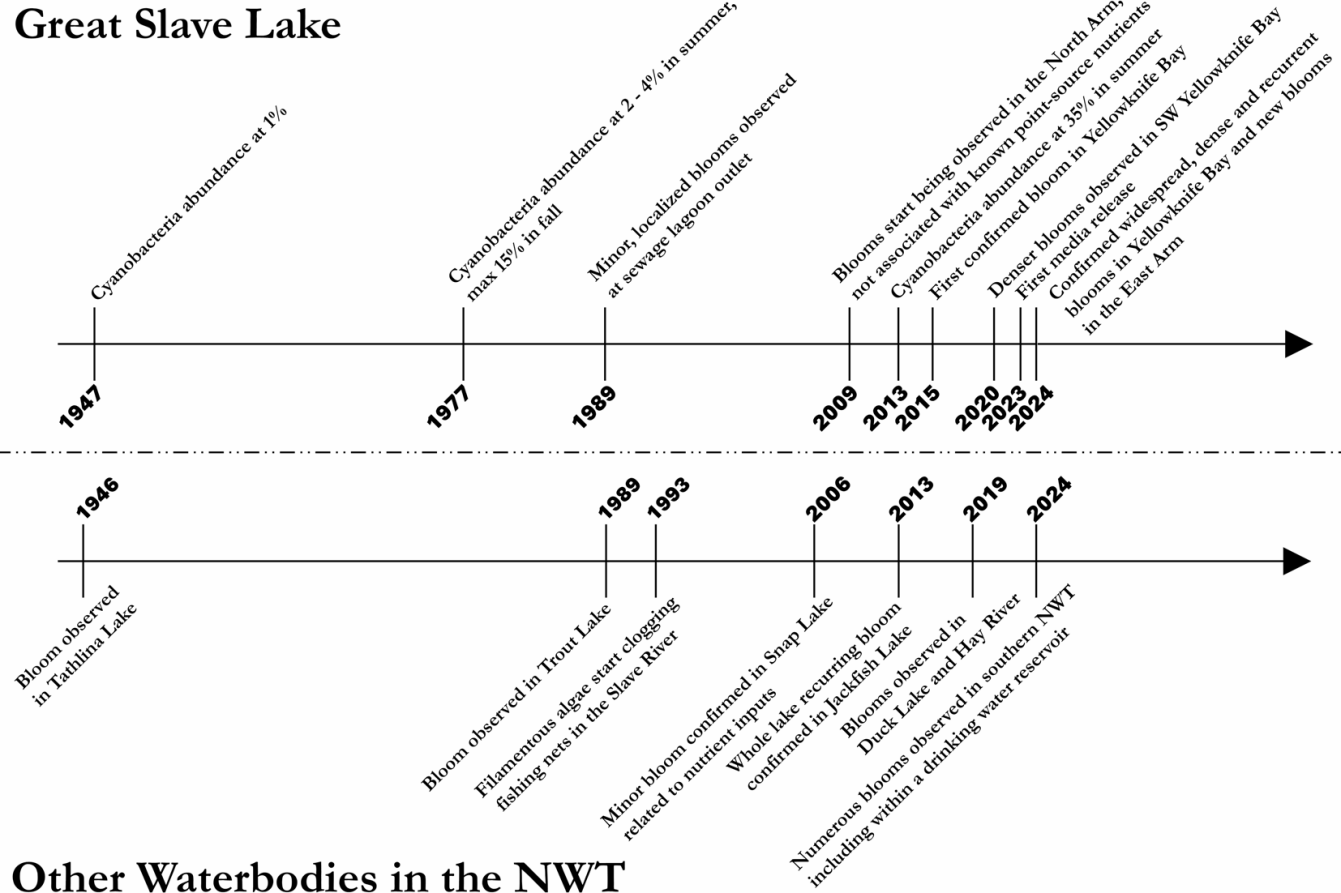
A summarized timeline of algal and cyanobacteria bloom developments in Great Slave Lake and the broader Northwest Territories (NWT).

**Fig. 3:**
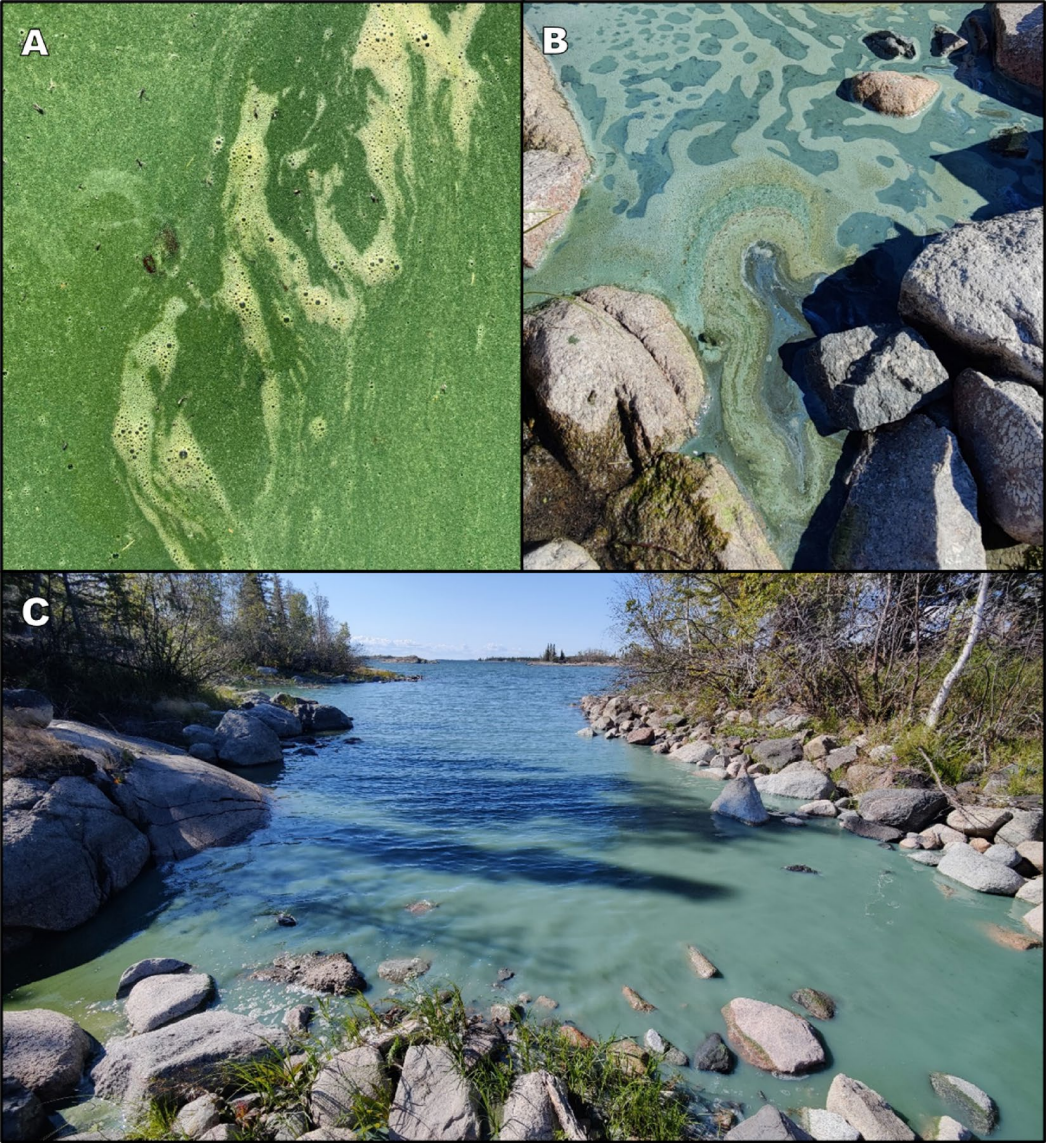
Photos of cyanobacteria blooms in Great Slave Lake, Northwest Territories, Canada. Photo A: September 4, 2020, in SW Yellowknife Bay (Photo: M. Fournier, reproduced with permission). Photos B and C: August 25, 2024, in SE Yellowknife Bay (Photo: J. Cederwall).

**Fig. 4:**
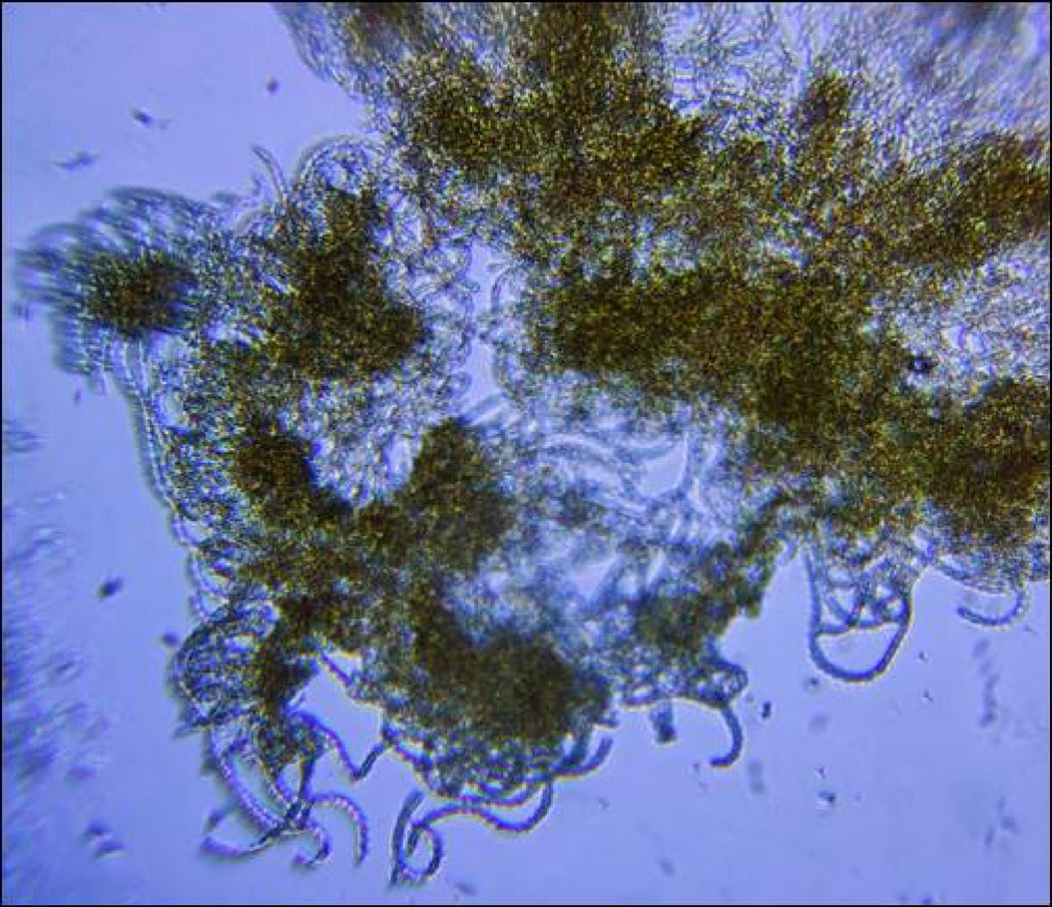
Micrograph of *Dolichospermum* sp. collected July 19, 2023 from a nearshore cyanobacteria bloom in Yellowknife Bay, Great Slave Lake, Northwest Territories, Canada. The sample was preserved with 1% acid Lugol’s solution and photographed using a field deployable Foldscope 2.0 at 140 × magnification on an Android OnePlus 9 camera (Photo: J. Cederwall).

Globally, more cyanobacterial blooms are being reported than ever before^42^ with growing concerns that climate change will continue to fuel this trend^43–45^. However, there remains debate as to whether these are true increases or attributable to improved detection, monitoring, and reporting^42^. By bridging Indigenous, local, and scientific knowledge sources, we confirm cyanobacterial blooms within Great Slave Lake are new and increasing (Figs. 2, 3 and 4), and do so within a jurisdiction that lacks a formalized algal bloom monitoring and reporting system. The recent increased occurrences of cyanobacterial blooms in this cold, deep, low-productivity lake are a striking departure from past conditions. The notable absence of generational Indigenous Knowledge relating to surface algal blooms, in such a large and culturally significant waterbody, supports the newness of these changes and indicates that historically, such blooms have not occurred. The absence of Indigenous Knowledge specific to mitigating the harmful effects of algal blooms in northern Canada contrasts regions such as southern Australia, where the local Aboriginal people were well aware of harmful algal blooms and employed a variety of traditional methods to reduce human health risks^43^. Due to the transient nature of nearshore cyanobacteria blooms and the scale and remoteness of Great Slave Lake, Indigenous and local land users were best positioned to make initial observations and describe patterns, which were then followed up collaboratively with scientific monitoring. Our bridged assessment confirms repeated and intensifying nearshore *Dolichospermum* spp. blooms across wide areas of Great Slave Lake. Although *Dolichospermum* is a genus known to sometimes produce cyanotoxins, including microcystins, anatoxins, saxitoxins, and cylindrospermopsins^44^, we did not detect microcystin toxins (> 0.2 µg/L DL) in Great Slave Lake.

The earliest record of *Dolichospermum* spp. in Great Slave Lake is from a comprehensive lake-wide scientific study conducted between 1944 and 1954 by Rawson^26^. Cyanobacteria were extremely rare, with only *Dolichospermum* (then known as *Anabaena)* occasionally found at ~ 1% relative abundance in phytoplankton hauls (Fig. 2), and no cyanobacterial blooms were reported^26^. With low abundance overall, only three *Dolichospermum* species were identified, including *D. lemmermannii*, *D. flos-aquae*, and *D. spiroides*, while *Microcystis* was absent^26^. It is noteworthy that between 1940 and 1981, Yellowknife Bay received nutrient-rich discharge from a sewage lagoon, which caused high *E. coli* levels^45^; however, no blooms were reported. Between 1975 and 1977, the relative abundance of cyanobacteria increased from 2–4% in the summer to a maximum of 15% in the fall, yet no blooms were reported^46^. Based on local knowledge gathered in this study, the earliest suspected blooms were observed in 1989 in an isolated region linked to local eutrophication, followed later by blooms in new locations starting in 2009. In June 2013, as part of gold mine regulatory monitoring, cyanobacterial relative abundance was found to reach 35% and was dominated by the filamentous *Lyngbya limnetica* and contained low but detectable concentrations of *Pseudanabaena* and *Dolichospermum;* again, no blooms were observed^47^. A suspected cyanobacteria bloom was documented in September 2013, where low density and characteristic surface streaking was seen within Yellowknife Bay, however, taxonomy was not confirmed^48^. There are no newspaper articles (Ryan Silke, Prince of Wales Northern Heritage Centre, *pers. comm.*), scientific articles, or regulatory reports describing any suspected cyanobacterial blooms before 2013.

In a survey of Indigenous Knowledge holders on Great Slave Lake, 87% (82 of 94 respondents) observed broadscale changes in Great Slave Lake, specifically water colour or cloudiness (68%), algae or vegetation (60%), foam or bubbles (56%), temperature (56%), and taste and smell (26%); the direction of > 77% of the changes described was an increase^39^. When asked about unusual “green scum / algal growth”, 57% (51 of 91 respondents) reported observing algae, seen predominantly in shallow water near islands, in the late summer. Indigenous communities on Great Slave Lake consistently prefer in-person engagement that allows for knowledge transfer through storytelling^39^, and written survey comments did not differentiate between characteristic cyanobacteria blooms, floating filamentous green algae mats, duckweed, attached periphyton or other algal growth. However, during conversational semi-structured interviews, we were able to focus on suspected cyanobacteria blooms through follow-up questions and discussion and were able to determine with them if their observations were likely cyanobacteria blooms or not.

Through a series of interviews with land users, we heard that the first suspected blooms on Great Slave Lake (~ 1989) were observed alongside other signs of local eutrophication within 1 km of the Fiddler’s Lake sewage lagoon discharge outlet, which is a remote, shallow area (< 4 m), sheltered by islands. It was not until the summer of 2009 that blooms were observed in new locations, not directly adjacent to known point source nutrients, east of Baker Island (Fig. 2; Mike Fournier, Environment and Climate Change Canada (ret.), *pers. comm.*). Collectively, land users were confident that, while there were other water quality changes observed, such as brownification and increased foam, there were no prior observations characteristic of cyanobacteria blooms elsewhere in Great Slave Lake. It is noteworthy that many land users were able to distinctly remember where and when they observed their first, as well as their most severe blooms, adding to the likelihood that this phenomenon had not been occurring before what is documented herein.

**Fig. 5:**
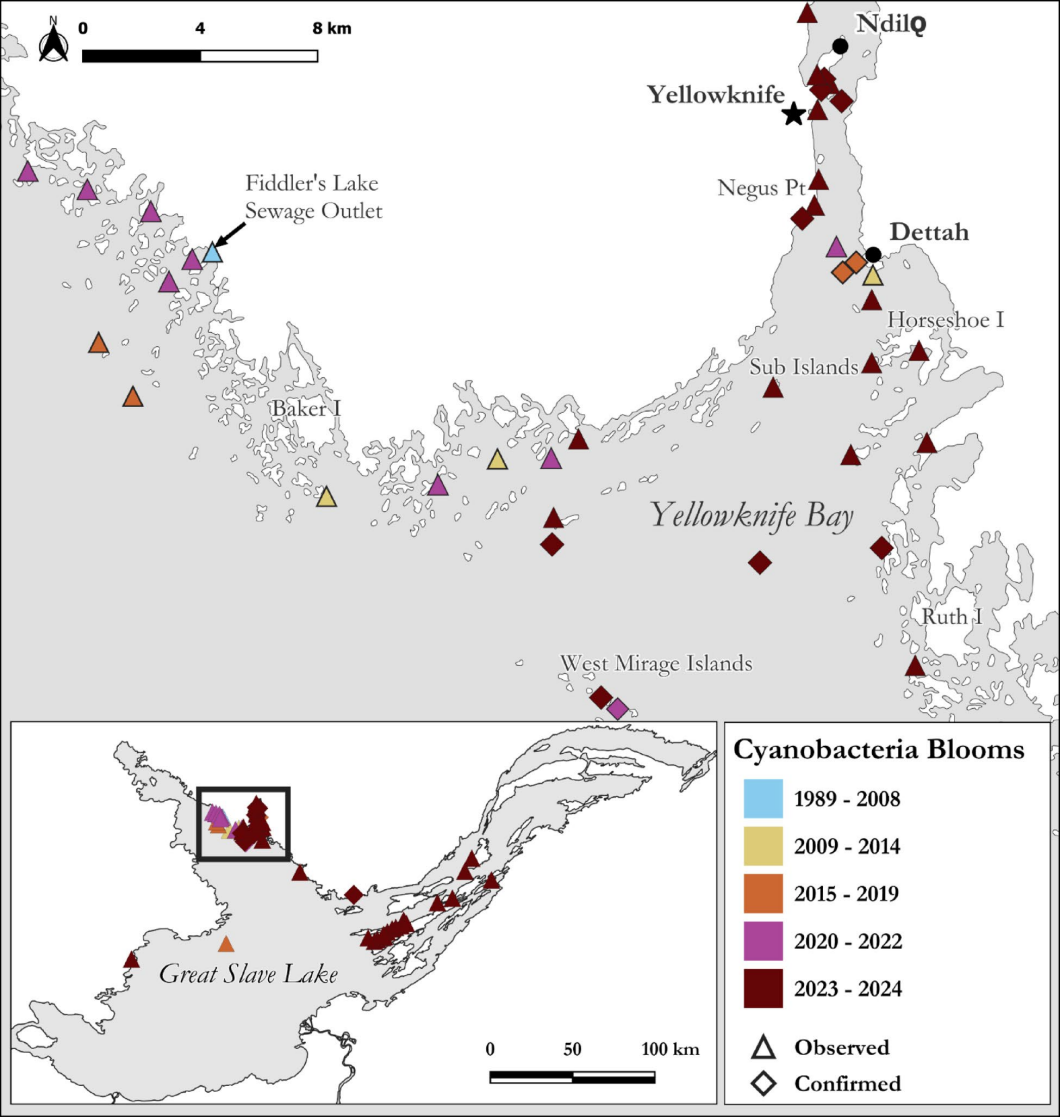
Cyanobacteria blooms in Great Slave Lake, Northwest Territories, Canada with a focus on the North Arm containing early hot spots (Yellowknife Bay and Baker Island). Observed blooms are those fitting characteristics of cyanobacteria surface blooms but were not sampled, while confirmed blooms were verified using light microscopy. No blooms were observed anywhere in Great Slave Lake before 1989. Note: Map created using QGIS 3.34.3 (https://www.qgis.org/).

Since 2009, local observations have continued, with Yellowknife Bay and the eastern vicinity of Baker Island being early hotspots (Fig. 5). For example, during the summer of 2014, visiting scientists reported finding it difficult to find clean water for drinking due to nearshore algal scums near their Baker Island field camp (Xinhua Zhu, Fisheries and Oceans Canada, *pers. comm.*). The first confirmed cyanobacteria bloom in Great Slave Lake was identified in 2015 as *D. lemmermannii*^49^. The bloom was visually estimated as low-density adjacent to the shoreline in Yellowknife Bay (John Chételat, Environment and Climate Change Canada, *pers. comm.*). In late August 2016, an aerial survey of the North Arm found two nearshore areas with unknown abundant phytoplankton. Subsequent aerial surveys were conducted within two weeks; however, the phytoplankton had dissipated following windy conditions. Some land users continued to see sporadic suspected cyanobacteria blooms in Yellowknife Bay and the nearby North Arm. These continued observations of suspected cyanobacteria blooms follow a 27% increase in Great Slave Lake’s primary production^50^ and a diatom regime shift from large, heavy *Aulacoseira islandica* to smaller, slower sinking, *Cyclotella* spp.^4^. Noticeably denser nearshore cyanobacteria blooms were seen by land users in late August–early September 2020 east of Baker Island in the North Arm, (Fig. 3a). On September 1, 2021, GNWT confirmed a nearshore *Dolichospermum spp.* bloom at the West Mirage Islands in the North Arm, following public concerns of dogs swimming in discoloured water. On August 11–15, 2022, a 7 km long unidentified bloom was visible in Sentinel-2 satellite imagery at the outlet of Fiddler’s Lake sewage lagoon system (Supplementary Fig. S9 and S10) but had dissipated by the next clear satellite pass over (10 days later).

Besides sporadic reports, cyanobacteria blooms in Great Slave Lake remained a relatively uncommon phenomenon. This changed in 2023, when in mid-July a cyanobacteria bloom appeared in Yellowknife Bay, following a period of warm weather and calm winds, where the surface water temperature reached 21 °C (the maximum recorded that year). This bloom was initially reported by the public as a suspected fuel spill, due to the unusual colour and foul smell. We subsequently identified this nearshore bloom as *Dolichospermum* spp. (2.7 × 10^5^ cyanobacteria cells/mL; Fig. 4). In response, the GNWT issued the first public bulletin for residents confirming cyanobacteria blooms in Great Slave Lake. During the same period, additional nearshore bloom sightings, characteristic of cyanobacteria, were reported by the public via social media posts, phone calls, email, and word of mouth. These blooms had occurred across the North Arm (20 and 45 km southeast of Yellowknife), as well as the Main Basin (125 km southwest of Yellowknife; Fig. 5). The Yellowknife Bay bloom was transient, dissipated with windy weather, which re-mixed surface waters into cooler deeper layers, which dropped the surface water temperature by 4 °C. No further blooms were reported in 2023; however, it should be noted that 60% of NWT’s population was ordered to evacuate because of severe wildfires, including several communities adjacent to Great Slave Lake (Yellowknife, Hay River, K‘atl’oDeeche First Nation, Ndilǫ, and Dettah). The evacuation lasted from mid-August to mid-September, when blooms would most likely occur. Further, the dense wildfire smoke prevalent in 2023 prevented comprehensive monitoring with satellite-based remote sensing.

In August 2024, following community concern, we confirmed a dense cyanobacteria bloom (11 × 10^5^ cyanobacteria cells/mL) dominated by *Dolichospermum* spp. (99%) which spanned > 1.5 km along Yellowknife’s shoreline. This bloom was transient, dispersed by strong winds within days of being first observed. However, more *Dolichospermum* spp. blooms were regularly observed throughout Yellowknife Bay and nearby islands until mid-September (Fig. 3b,c). The location of blooms appeared to be dependent on wind direction with the density of the bloom driven, at least in part, by wind strength. On calm days, these blooms extended from the shoreline to 1 km offshore. Of particular note, in early September 2024, cyanobacteria blooms were observed for the first time in the East Arm. These blooms were widespread nearshore among islands of the large archipelago (100–130 km southeast of Yellowknife (Shawne Kokelj, GNWT, *pers. comm.*; Fig. 5). We subsequently confirmed a separate cyanobacteria bloom (4.5 × 10^5^ cyanobacteria cells/mL) dominated by *Dolichospermum* spp. (93%) and *Pseudanabaena* spp. (7%) along the north shore of the East Arm, approximately 80 km east of Yellowknife (Fig. 5).

Cyanobacteria blooms in Great Slave Lake have been consistently dominated by *Dolichospermum* spp. Similarly, blooms of this genus (*D. lemmermannii*) have been reported in nearshore areas of other large, oligotrophic lakes, such as in the western basin of Lake Superior^51^, as well as in its deeper north shore^52^. Likewise, *D. lemmermannii* blooms have been observed in Russia’s huge oligotrophic Lake Baikal^53^. In Great Slave Lake, we did not detect microcystins (DL: 0.2 µg/L) associated with cyanobacteria blooms in either 2023 or 2024. Similarly, no microcystins (> 0.2 µ/L) were detected in the earliest sampled cyanobacteria blooms in 2015^49^. Likewise, microcystins were not detected in *D. lemmermannii* blooms in Lake Superior, and instead, only anabaenopeptin was occasionally found^51^. The *D. lemmermannii* blooms in Lake Baikal contained low levels of saxitoxins^53^, and although microcystins were not analyzed, it was later determined that some strains of *Dolichospermum* found in Lake Baikal are capable of microcystin production^54^. As *Dolichospermum* and *Pseudanabaena* genera are potentially toxigenic, screening for the full suite of cyanotoxins is required to assess which cyanotoxin genes are present and learn what may trigger cyanotoxin production in large lakes^55^. When cyanobacteria scums are concentrated by wind and currents, densities can greatly exceed Canadian recreational water quality guidelines (> 5.0 × 10^4^ cells/L)^41^ and World Health Organization (WHO) alert levels^56^, indicating potential cyanotoxin risk. However, unlike whole-lake blooms, nearshore blooms—such as those observed in Great Slave Lake—pose localized risks and may also negatively affect cultural and recreational activities^41^.

Warming of oligotrophic lakes can confer advantages for cyanobacteria with certain functional traits. In smaller oligotrophic lakes within temperate regions of the Northern Hemisphere, climate change is believed to have increased cyanobacteria blooms^57,58^, often independent of anthropogenic point source nutrient inputs. Members of the cyanobacterial order Nostocales, including *Dolichospermum* spp., can form akinetes (resting stages), which allows for both their successful overwintering and improved phosphorus gathering from sediments until conditions are optimal^59^. Many expect these species to succeed in a warming climate as temperatures in temperate and subarctic waters increase towards the optimum temperature for growth^15,60^. Warming temperatures combined with calmer winds are increasing the thermal stratification of Great Slave Lake^4^. These conditions will facilitate *Dolichospermum*’s life cycle including their gas vesicle-driven buoyancy regulation^61,62^ and will likely promote surface scums to form more frequently.

While we propose climate change is a primary driver for the increased proliferation of cyanobacteria observed blooms in Great Slave Lake, nutrients remain a key constituent for influencing and enabling blooms. Across the Northern Hemisphere, long-term atmospheric deposition of anthropogenic nitrogen exacerbated phosphorus limitation in oligotrophic and mesotrophic lakes^63,64^. Typical point-source nutrient pollution, such as the phosphorus-rich effluent from sewage lagoons, can locally influence their receiving waters. The earliest suspected blooms in Great Slave Lake were associated with effluent from the Yellowknife sewage lagoon (Supplementary Fig. S11–S15 and Table S2). Under a changing climate, external nutrient inputs can increase. From 1948 to 2012, annual precipitation in the Great Slave Lake region increased by 24%, potentially adding allochthonous nutrients^65^. Recent weather has been highly variable, breaking previous temperature and rainfall records, highlighting the influence of variability, not just long-term trends. Water levels on Great Slave Lake have dramatically fluctuated over the last five years, ranging from the highest levels on record in 2020 to the lowest levels in 2023–2024 (based on 82 years of gauged monitoring (Water Survey of Canada, 2025, station ID: 07OB001). For example, in the summer of 2020, exceedingly high precipitation resulted in a pronounced increase in water flow and sediment load through Slave River, delivering a visible nutrient-rich sediment plume substantially further into the North Arm and the East Arm archipelago regions (Supplementary Fig. S16). Further, warmer temperatures combined with extreme drought cycling have led to unprecedented wildfire seasons (2014, 2023, 2024) producing dense and frequent smoke over the region. This smoke and ash can provide accessible nutrients to fuel blooms, particularly in phosphorus-limited oligotrophic lakes^66^. Lastly, the continued thawing of permafrost can release accessible phosphorus and iron^67,68^ which are known to promote blooms for nitrogen-fixing taxa, like *Dolichospermum* spp.^18,69–72^.

Our study was limited by the lack of established, standardized lake phytoplankton monitoring programs. Using opportunistic scientific data, Indigenous and local knowledge, we have documented the emergence and increase of northern cyanobacteria blooms. However, Indigenous and local knowledge is inherently focused on areas and times accessed and used by people. Satellite-based high-resolution remote sensing, calibrated with in-situ samples, can assist in quantifying and tracking future blooms, but these data are also hampered by limited visibility during periods of heavy wildfire smoke. Inconsistencies in northern regulatory requirements between municipal and industrial wastewater have contributed to scientific data gaps, with no required testing of phytoplankton taxonomy or cyanotoxins required for treated drinking water municipal water sources, or water bodies receiving municipal sewage. Finally, the lack of cyanobacteria and cyanotoxin testing laboratory capabilities across Canada’s northern territories limited testing and delayed response times.

There is growing concern about the potential spread of harmful algal blooms to new locations, particularly if accompanied by toxins^73,74^. The unexpected and increasing occurrences of cyanobacteria blooms in Great Slave Lake raise questions about whether similar blooms are already present but have remained undetected in other remote northern waterbodies, or whether new blooms may emerge. Identifying which waterbodies, or types of waterbodies, are at greatest risk for blooms and toxin production is an emerging priority. While more data are required for implementation, machine learning algorithms (such as artificial neural networks and random forest models), or generative-style Bayesian models (networks and spatiotemporal) could provide short-term predictions of these bloom events^75–78^.

While our study focused on Great Slave Lake, broad-scale climate-related changes and nutrient inputs appear to be driving environmental and ecological shifts observed across many water bodies in the NWT. Indigenous and local knowledge has highlighted an overall increase in ecosystem productivity, including cyanobacteria, algae, periphyton, aquatic plants, and iron bacteria scums. From our combined literature review and knowledge holder interviews, algal and cyanobacterial blooms have been observed in water bodies across the Taiga Plains and Taiga Shield, including in drinking water reservoirs (see Supplementary Information 6 for more details). Combined, these observations and anticipated environmental changes highlight the need for adaptive management strategies that include using Indigenous, local, and scientific knowledge systems synergistically.

## Conclusion

Climate change is accelerating environmental change in terrestrial, marine, and freshwater ecosystems across the Arctic and subarctic^79–81^. Here, we present a striking example of the emergence and rapid increase of cyanobacterial blooms on Great Slave Lake over the past 15 years. Despite its northern latitude, short growing season, oligotrophic status, great size, and cold, deep waters, Great Slave Lake is also vulnerable to the impacts of climate change and nutrient inputs. While it remains unclear if we are yet at an ecological tipping point, the rapid emergence and increase of northern cyanobacteria blooms can be seen as harbingers of environmental change^82,83^. The unprecedented increase in scale and frequency of recent northern blooms should serve as a call to action and highlight the need to respectfully bridge knowledge systems to collectively address longstanding research and monitoring shortcomings in one of Canada’s northern “great” lakes. Given the ecological and public health threats posed by cyanobacteria blooms, many governments around the world have instated coordinated monitoring, management, and public communication processes. As climate change continues, implementing surveillance programs designed to monitor and manage cyanobacteria blooms in all three of Canada’s northern territories becomes ever more pressing^84^, and should be seen within a climate change adaptation context. Research is required to better understand the drivers of these widespread and increasing cyanobacteria blooms in Great Slave Lake and other northern waterbodies, while aiming to reduce anthropogenic nutrient inputs. Over 70 years ago, Rawson^85^, lead researcher of Great Slave Lake’s most comprehensive scientific study to date, flagged the severe knowledge gaps across North America’s numerous northern lakes and highlighted the need for intensive limnological study, including winter sampling and investigating warming waters. We echo this sentiment and recommend that these efforts be respectfully conducted with continued sharing and cooperation among Indigenous, local, and science knowledge holders.

## Electronic supplementary material

Below is the link to the electronic supplementary material.

Supplementary Material 1

Supplementary Material 2

## Data Availability

All scientific data in this manuscript (aerial surveys, phytoplankton taxonomy, and microcystin toxin analysis) are available in the supplementary files. The observations of Great Slave Lake cyanobacteria blooms generated and analyzed during the current study are available in supplementary files; information has been anonymized unless otherwise agreed to by interviewees and noted in the paper. Standard water chemistry parameters are available online at Mackenzie DataStream (https://doi.org/10.25976/3ogv-7n39).
