# Supplementary material for: Rapidly increasing cyanobacteria blooms in the subarctic Great Slave Lake: observations from Indigenous, local, and scientific knowledge
Source: Sci Rep. 2025 Jul 8;15:24492. doi: 10.1038/s41598-025-07432-5 (PMC12238587; doi:10.1038/s41598-025-07432-5)
Supplement: Supplementary file 1 — Supplementary Material 1 [file 41598_2025_7432_MOESM1_ESM.pdf]

Supplementary Information for

Rapidly increasing cyanobacteria blooms in the subarctic Great Slave Lake: observations from Indigenous, local, and scientific knowledge

Jeffrey Cederwall[a1]* and Peter A. Cott[a]*

[a] Government of the Northwest Territories, Department of Environment and Climate Change, 5102 – 50th Ave. Yellowknife, NT, X1A 2L9, Canada

[1] Present address: Canada Water Agency, 5019 – 52nd St. Yellowknife, NT, X1A 1T5, Canada

*Corresponding author email: Jeffrey.Cederwall@cwa-aec.gc.ca

*Corresponding author email: Pete_Cott@gov.nt.ca

Table of Contents

Supplementary Information 1: Great Slave Lake water chemistry....2
Supplementary Information 2: Methods....3
Supplementary Information 2a: Great Slave Lake community survey....3
Supplementary Information 2b: Semi-structured interview questions....5
Supplementary Information 2c: Great Slave Lake aerial survey....6
Supplementary Information 2d: Phytoplankton taxonomy....10
Supplementary Information 2e: Microcystin toxin screening....11
Supplementary Information 3: City of Yellowknife Fiddler's Lake sewage lagoon....12
Supplementary Information 4: Slave River sediment plume....21
Supplementary Information 5: Observed water quality changes across the NWT....22
Great Slave Lake sub-basin....22
Southwest Northwest Territories in the Taiga Plains....22
Northern Northwest Territories in the Taiga Shield....23
Western Northwest Territories in the Mackenzie Mountains and Mackenzie Delta....23

Supplementary Information 1: Great Slave Lake water chemistry

Physiochemical parameter	North Arm	East Arm
pH	7.86 (7.23–8.38)	7.89 (7.19–8.39)
Specific Conductance (uS/cm)	224 (73.3–251)	205.5 (38.1–243)
Alkalinity, as CaCO3 (mg/L)	76.8 (26.6–88.2)	72.3 (13.7–85.4)
Hardness, as CaCO3 (mg/L)	98.5 (30.8–121)	91.85 (18.9–111)
Turbidity (NTU)	1.46 (0.61–3.79)	0.435 (0.15–1.8)
Dissolved Organic Carbon (µg/L)	5.3 (4.4–7.5)	4.7 (2.2–6.6)
Total Nitrogen (µg/L)	280 (230–1800)	270 (210–470)
Total Phosphorus (µg/L)	7 (2–13)	5 (2–16)
Total Iron (µg/L)	40 (16–100)	9 (5–48)

Table S1: Great Slave Lake physicochemical surface data collected by the Government of the Northwest Territories – Department of Environment and Climate Change for 2023–2024, reported as median with range. Data available online at Mackenzie DataStream https://doi.org/10.25976/3ogv-7n39.

Supplementary Information 2: Methods

Supplementary Information 2a: Great Slave Lake community survey

Government of Northwest Territories / Gouvernement des Territoires du Nord-Ouest

INTRODUCTION

The Government of the Northwest Territories (GNWT) Department of Environment and Climate Change (ECC), in partnership with the Department of Fisheries and Oceans Canada (DFO) and Arctic Research Foundation (ARF), are inviting members of the **XXX-INDIGENOUS GOVERNMENT-XXX** to take part in a survey that will provide input to the development of a research and monitoring program for Great Slave Lake.

The purpose of the survey is to hear directly from community members about:

- The changes being observed on and around Great Slave Lake
- The research and monitoring questions that community members have
- Environmental concerns regarding Great Slave Lake
- The research and monitoring priorities of communities located around Great Slave Lake
- How the GNWT and our partners can best collaborate and work as community partners
- Best engagement and communication practices

This survey is available for all members of the **XXX-INDIGENOUS GOVERNMENT-XXX** and is also being distributed in other communities located around Great Slave Lake. It should take about 1 hour to complete and consists of 32 questions. Each person who completes the survey will receive **$XXX** from the **XXX-INDIGENOUS GOVERNMENT-XXX**.

By participating in this survey, you are agreeing to have your survey responses anonymously included in a report that will be made public. Your name and personal informational will NOT appear in results reporting. The survey data will be co-owned with participating communities and shared anonymously more widely through a report that will be made publicly available following the completion of the survey in all participating communities. The anonymous survey results may also be used in existing and future related research and monitoring program planning, reporting, and communications materials including community engagement sessions, workshops, and conferences by the listed partner organizations.

Please contact **XXX-NAME OF LOCAL COMMUNITY ENGAGEMENT COORDINATOR-XXX** with any general questions about this survey.

Fig. S1: Great Slave Lake Research and Monitoring Indigenous Community Survey participant disclaimer, with overview of data co-ownership, and anonymous data use.

PART 1: Changes to the environment over time

1. Have you noticed changes in the quality of the water in Great Slave Lake?

☐ The color and clarity / cloudiness of the water ☐ More ☐ Less
☐ Algae or Vegetation growth ☐ More ☐ Less
☐ Foam or bubbles on the surface ☐ More ☐ Less
☐ Taste or smell
☐ Temperature ☐ Warmer ☐ Colder
☐ No observed changes
☐ Other (please identify below)

Which of these changes has you most concerned and why? How long have you noticed these changes?

2. Have you noticed any unusual growth of green scum (algae) on Great Slave Lake?

☐ Yes ☐No

If you answered yes, where and when have you noticed the algae?
☐ Near islands
☐ Shallow water
☐ Deeper water
☐ Other locations (please specify below)
☐ Spring
☐ Early summer
☐ Late summer
☐ Fall
☐ Under ice
☐ Other (please specify below)

Fig. S2: Great Slave Lake Research and Monitoring Survey observational questions 1 and 2.

Supplementary Information 2b: Semi-structured interview questions

- Have you noticed any algal blooms on the lake before? Were these surface scums of algae like spilled paint, mixed into the water column or attached to rocks?
    - If yes, attempts were made to try distinguishing between potential cyanobacteria blooms and filamentous green algae, duckweed, microbial mats, bacterial sheen, iron bacteria, wildfire ash, and pollen. In some cases, photos of confirmed Great Slave Lake cyanobacteria blooms were provided.
    - If no suspected cyanobacteria blooms were observed, questions focused on the interviewees' use of Great Slave Lake to understand areas and times not seen. (e.g. How long have you been regularly on Great Slave Lake? Which areas do you typically use).
- How would you describe it? What did it look like (colour, smell, consistency, floating vs non-floating)?
- What did it look like? What colour was it? Did it have a smell? How large of an area was covered? Could you see the lake bottom nearshore? Did you notice any dead wildfire or fish in the area?
- Would this clump to a stick and look like wet wool or coat it like paint?
- When did you see this (date)?
- Where did you see this (area of Great Slave Lake or inland lake)?
- Have you seen this before?
- Do you have any photos?
- When/ where was the first time you observed a surface scum of algae?
- When/ where was the thickest/ largest amount of surface scum algae you saw?
- What did it look like? What colour was it? Did it have a smell? How large of an area was covered? Could you see the lake bottom nearshore? Did you notice any dead wildfire or fish in the area?
- Are there specific areas you noticed this more often?
- Are there other unusual water quality observations you noticed in the waters of Great Slave Lake?
- What about it is unusual? When and where did you see this?
- Is there anything else you would like to share?

Supplementary Information 2c: Great Slave Lake aerial survey

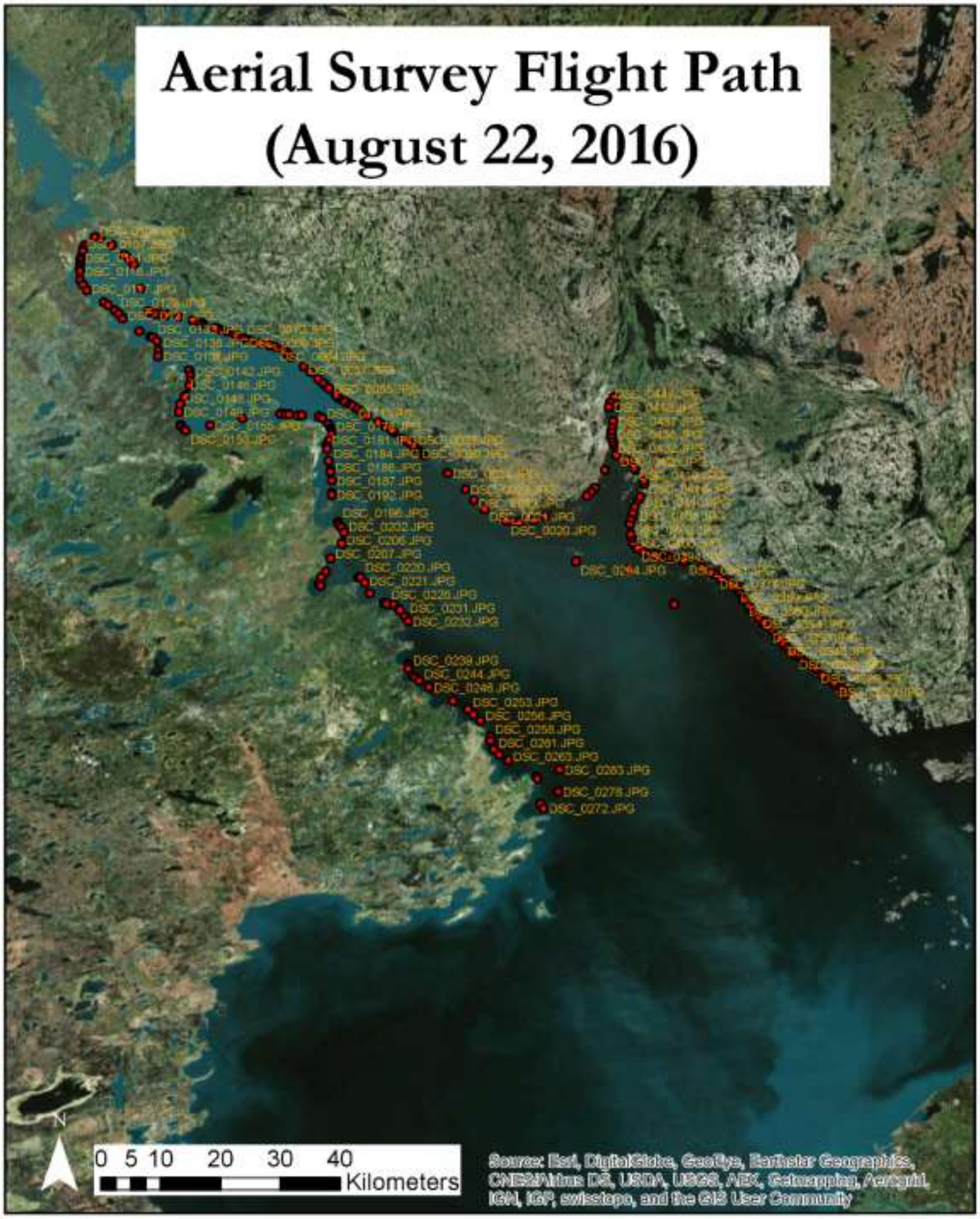

Fig. S3: Flight path and photo locations of the August 22, 2016, Great Slave Lake - North Arm aerial survey for surface algal blooms. Note: Aerial surveys were conducted in partnership by Government of the Northwest Territories – Department Environment and Climate Change with Environment and Climate Change Canada.

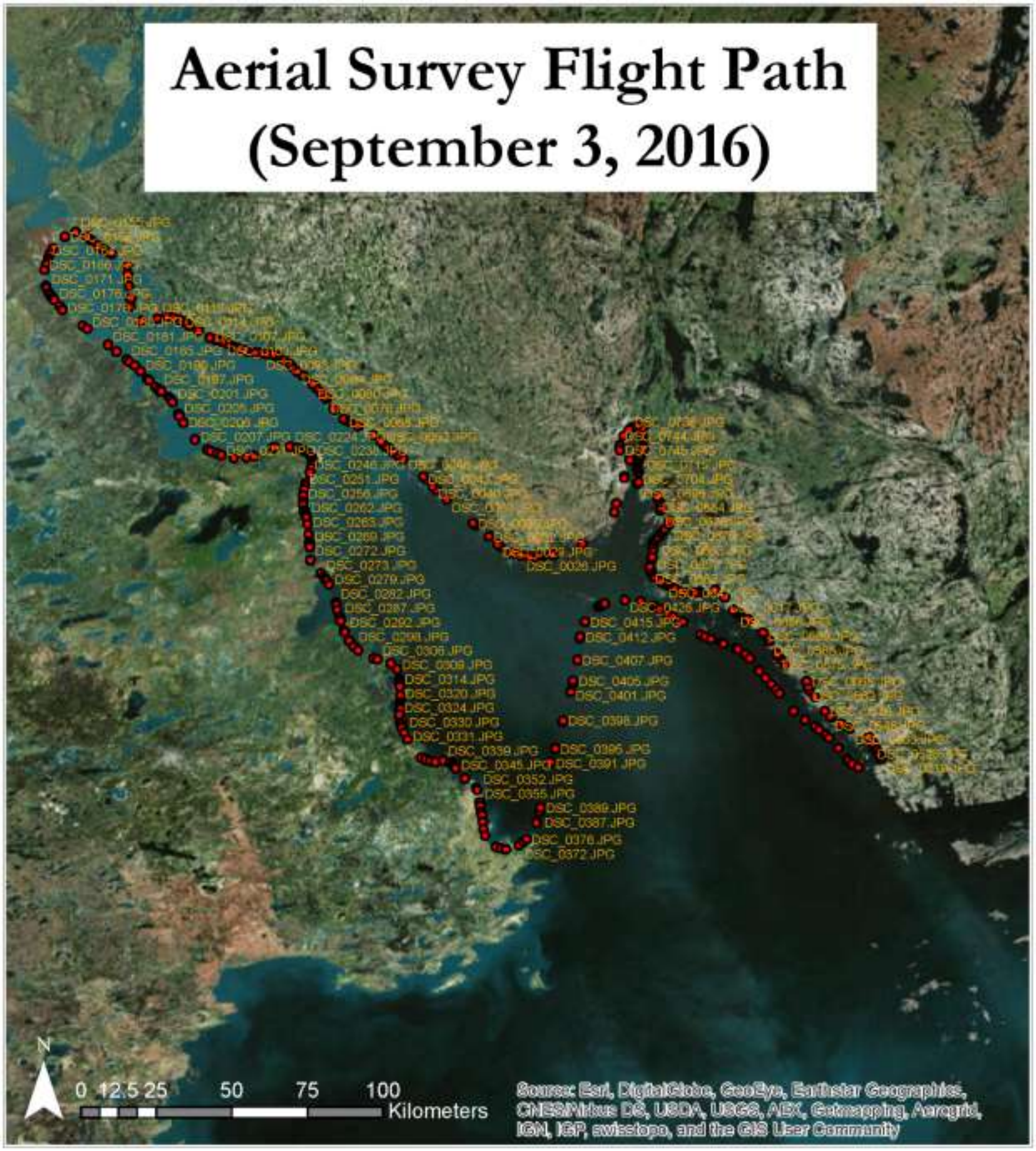

Fig. S4: Flight path and photo locations of the September 3, 2016, Great Slave Lake - North Arm aerial survey for surface algal blooms. Note: Aerial surveys were conducted in partnership by Government of the Northwest Territories – Department Environment and Climate Change with Environment and Climate Change Canada.

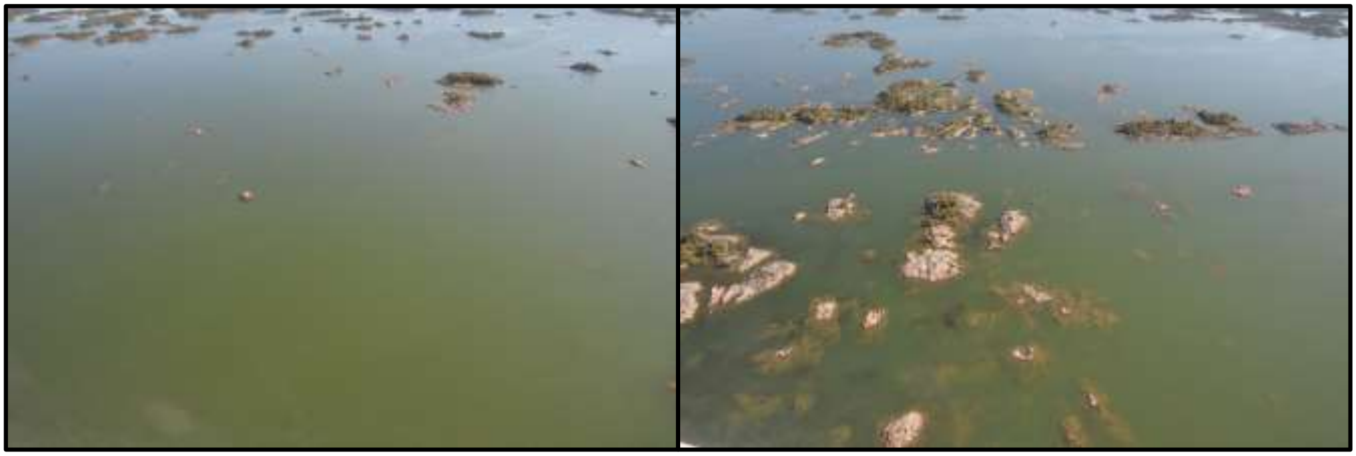

Fig. S5: Examples of Aug 22, 2016 North Arm aerial images classified as high phytoplankton abundance. A) DSC_0022 and B) DSC_0023. Photos: Stefan Goodman, Government of the Northwest Territories – Department of Environment and Climate Change.

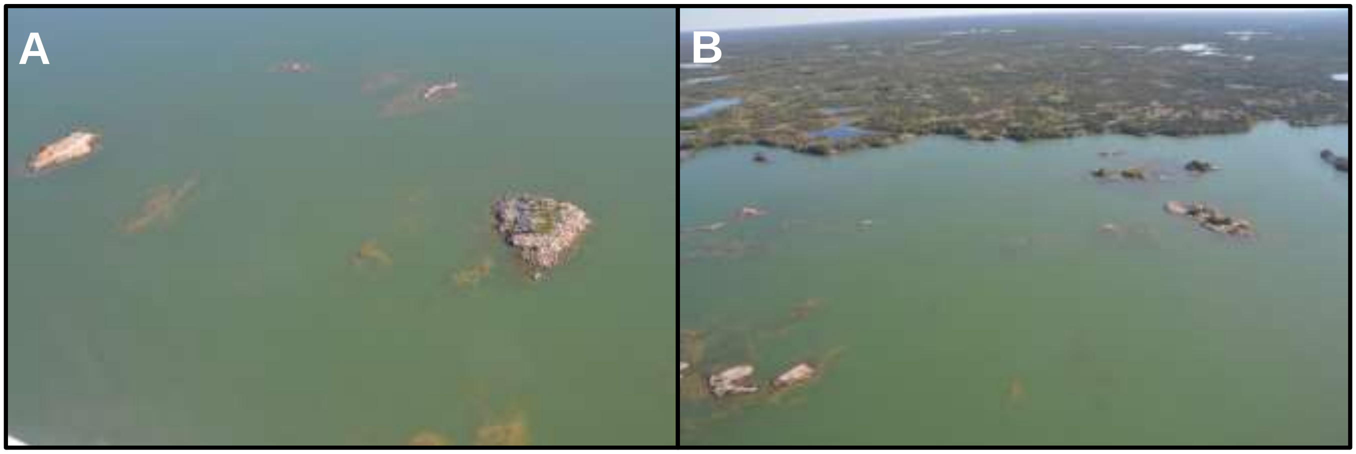

Fig S6: Examples of Aug 22, 2016 North Arm aerial survey images classified as medium phytoplankton abundance. A) DSC_0021 and B) DSC_0036. Photos: Stefan Goodman, Government of the Northwest Territories – Department of Environment and Climate Change.

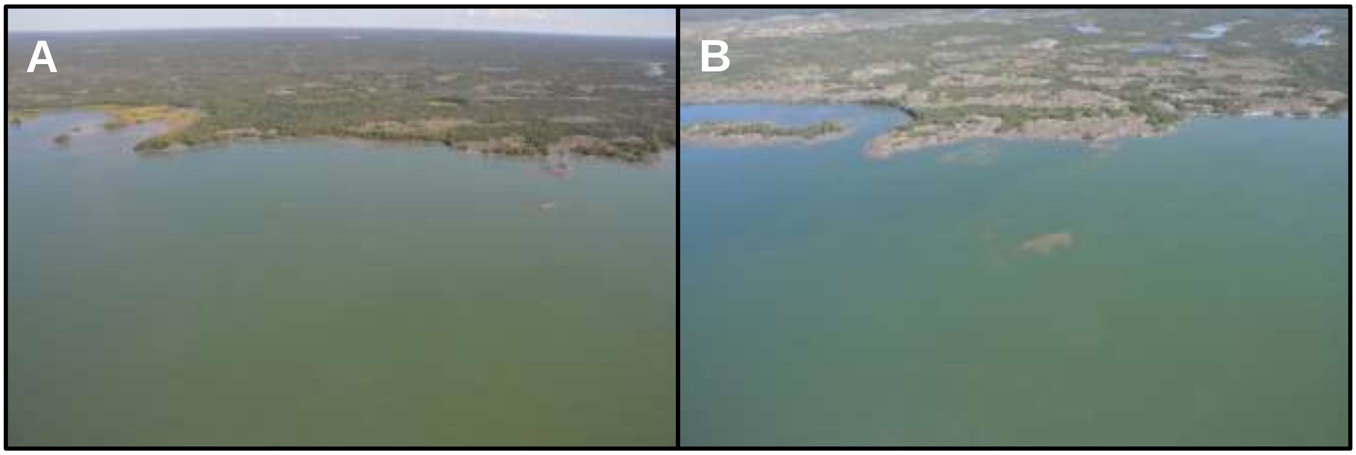

Fig S7: Examples of Aug 22, 2016 North Arm aerial images classified as low phytoplankton abundance. A) DSC_0329 and B) DSC_0337. Photos: Stefan Goodman, Government of the Northwest Territories – Department of Environment and Climate Change.

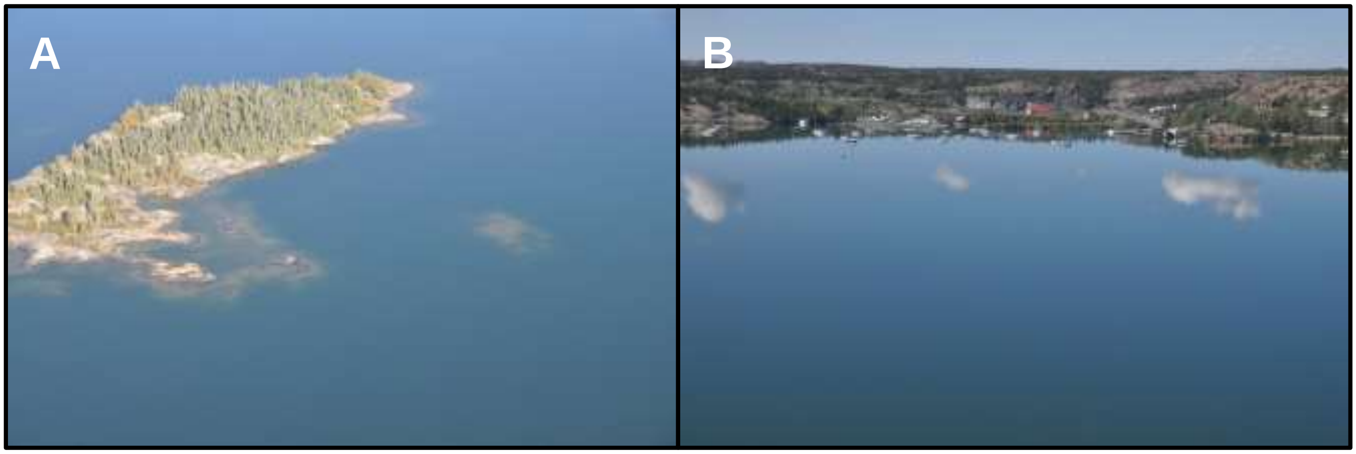

Fig. S8: Examples of Aug 22, 2016 North Arm aerial images classified as clear (no visible phytoplankton abundance). A) DSC_0016 and B) DSC_0448. Photos: Stefan Goodman, Government of the Northwest Territories – Department of Environment and Climate Change.

Supplementary Information 2d: Phytoplankton taxonomy

ALS Environmental Laboratory (Winnipeg, MB) uses a low-volume Utermöhl method based on APHA 10200[1]. The ALS Environmental lab method code is E921A: blue-green algae.

Phytoplankton samples are concentrated following APHA 10200C. Typically, 1 mL of Lugol's preserved sample is added to a 5 mL cylindrical settling chamber containing a thin, clear glass bottom. The settling chamber is then filled to the top with 4 mL of deionized, UV treated water and allowed to settle overnight.

Settled phytoplankton are identified and enumerated to genus level using an inverted microscope at 100x and/or 400x following APHA 10200E and APHA 10200F. Typically counts are done using "field counting" where the field is the full field of view at 100x magnification and then repeated 10 times spaced out across the whole settling chamber. Field counts may be done at 400x magnification depending on cell concentration and their visibility at 100x magnification. Occasionally, if large amounts of phytoplankton cells are present, a Whipple grid may be used and repeated 10 times spaced out across the whole settling chamber. Following field counts, a scan of the whole chamber is done to count any species not counted in the fields, or to count larger colonies that were too big for the area of the field. Samples with low concentrations of phytoplankton may count the whole chamber instead of performing field counts. The reported detection limit is 1 cell/ mL, as the whole chamber is scanned.

References

1. Standard Methods Committee of the American Public Health Association, American Water Works Association, and Water Environment Federation. 10200 plankton In: Standard Methods for the Examination of Water and Wastewater. Lipps WC, Baxter TE, Braun-Howland E, editors. Washington DC: APHA Press. https://doi.org/10.2105/SMWW.2882.207

Supplementary Information 2e: Microcystin toxin screening

Total microcystins were screened at the ALS Environmental Laboratory (Winnipeg, MB, CANADA) using a EnviroLogix QualiTube Kit for Microcystin (CAT. EP022) with a pre-sonication step to lyse cells. This competitive Enzyme-Linked ImmunoSorbent Assay (ELISA) kit does not distinguish between microcystin congeners and includes Microcystin-LR, Microcystin-LA, Microcystin-RR, Microcystin-YR, and Nodularin. Following the assay, colour development was measured via spectrophotometry at 450 nm wavelength. Sample batches were analyzed following ALS standard protocols which contained the following laboratory control samples: Method Blank (analyte-free matrix), Laboratory Control Sample (analyte-free matrix that has been fortified/spiked with test analytes at known concentration), and a Matrix Spike (randomly selected intra-laboratory replicate sample that has been fortified/spiked with test analytes at known concentration). The detection limit is 0.2 µg/L.

Supplementary Information 3: City of Yellowknife Fiddler's Lake sewage lagoon

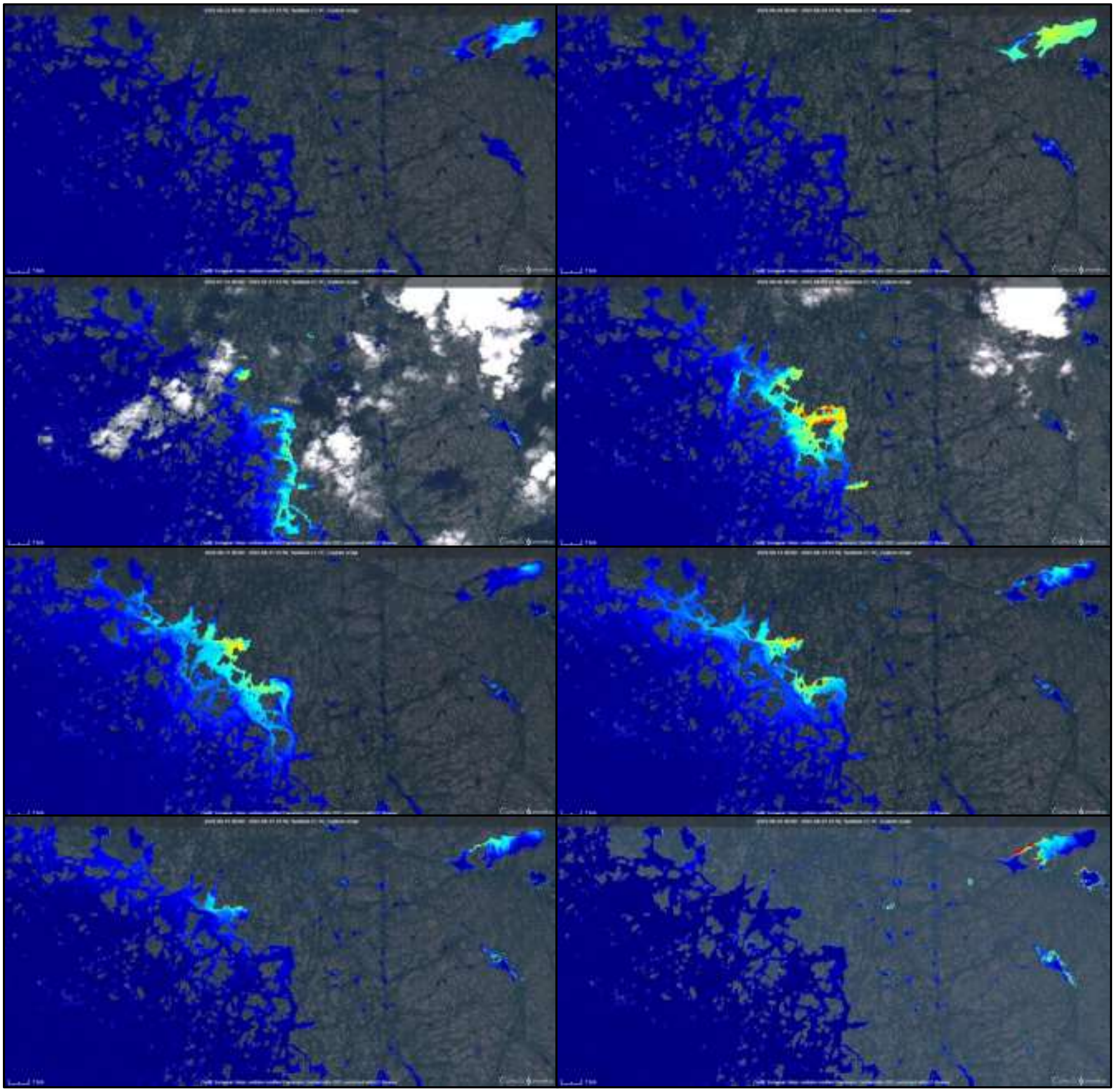

Fig. S9: Outlet of Fiddlers Lake Sewage Lagoon in Great Slave Lake visualized using Sentinel-2 satellite with the Maximum Peak Height Bloom Index collected on the following dates in 2022: A) Jun. 22, B) Jul. 29, C) Jul. 31, D) Aug. 5, E) Aug. 11, F) Aug. 13, G) Aug. 15, H) Aug. 25. Credit: European Space Agency; contains modified Copernicus Sentinel data [2025] processed by Sentinel Hu

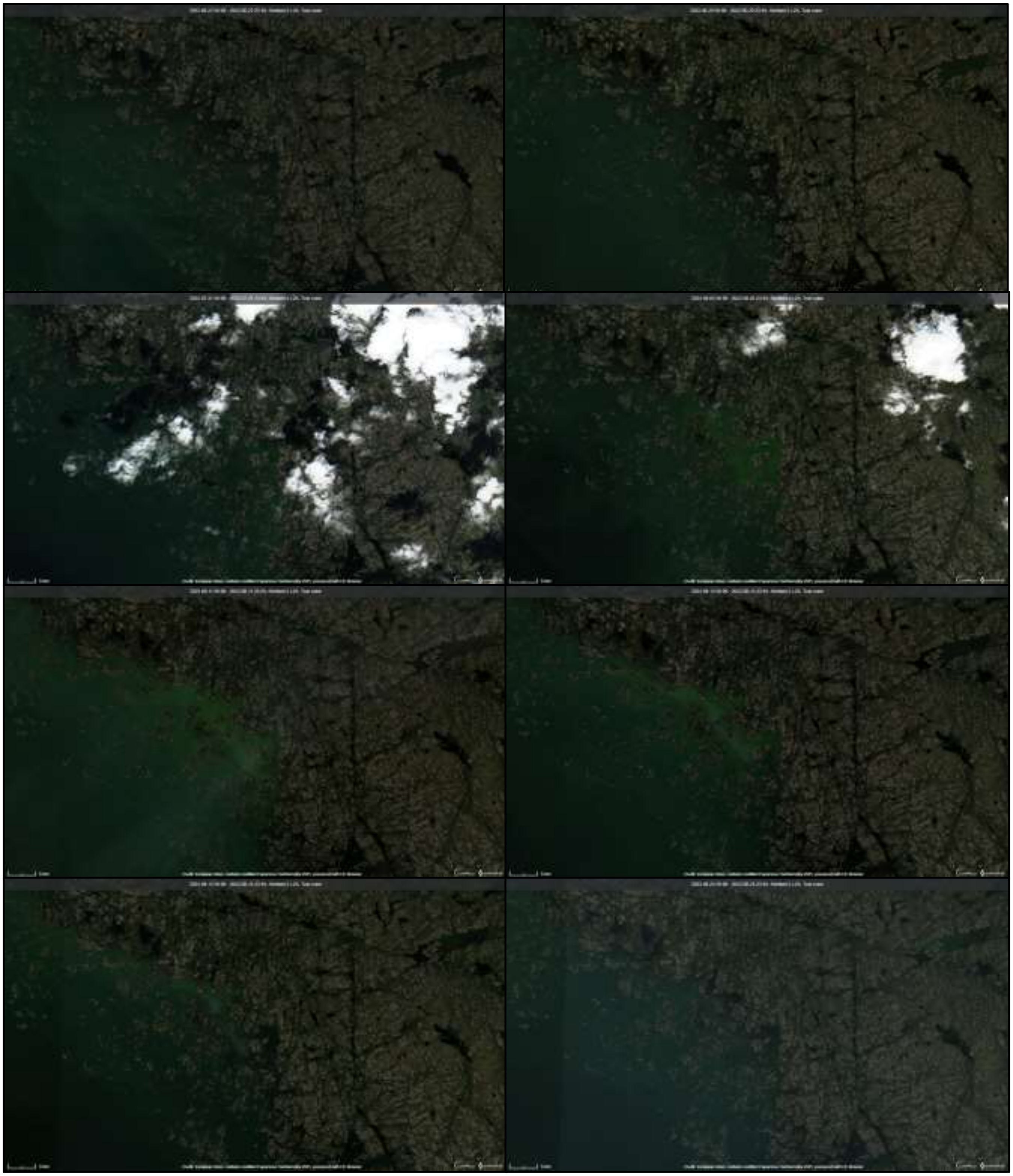

Fig. S10: Outlet of Fiddlers Lake Sewage Lagoon in Great Slave Lake using Sentinel-2 in true colour collected on the following dates in 2022: A) Jun. 22, B) Jul. 29, C) Jul. 31, D) Aug. 5, E) Aug. 11, F) Aug. 13, G) Aug. 15, H) Aug. 25. Credit: European Space Agency; contains modified Copernicus Sentinel data [2025] processed by Sentinel Hub.

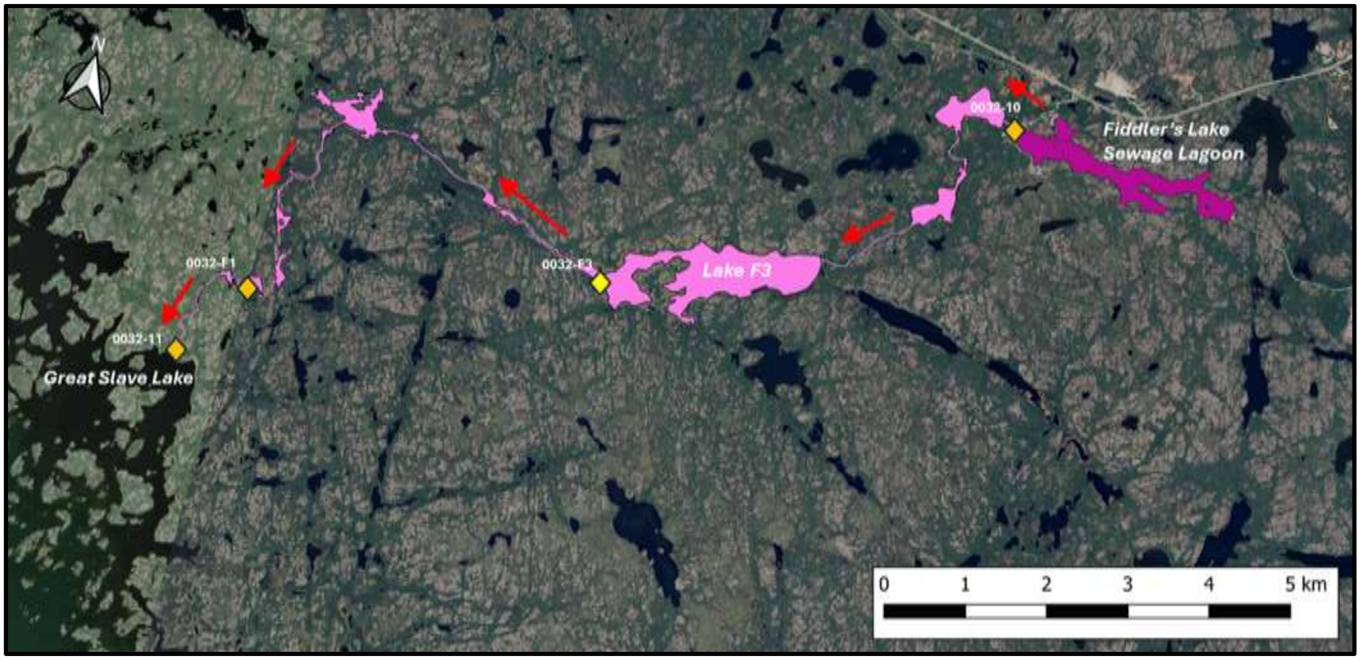

Fig. S11: Effluent flow path from the City of Yellowknife's Fiddler's Lake Sewage Lagoon (purple colour) through the series of natural wetlands and lakes (pink colour) before discharge into Great Slave Lake. Red arrows highlight the direction of the primary flow path. Regulatory monitoring sites are noted with diamonds (coloured yellow to highlight the effluent compliance point and orange for additional monitoring sites). Basemap: Google Satellite (obtained through QGIS XYZ Connection), Map data ©2025 Google.

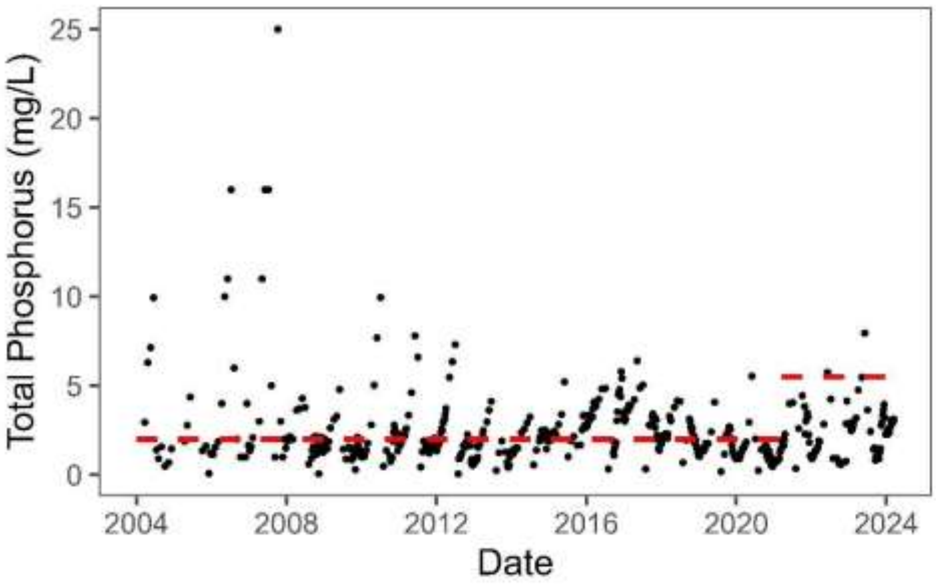

Fig. S12: Total phosphorus at the outlet of Lake F3, downstream of Fiddler's Lake Sewage Lagoon. The red dashed lines denote the Water License's maximum grab samples effluent objectives and standards (2 mg/L from 2002-2021; 5.5 mg/L beginning in 2022). Data source: City of Yellowknife's Water License Reports (N1L4-0032, N1L3-0032, MV2009L3-0007, MV2021L3-0003) available online at Mackenzie Valley Land and Water Board Public Registry (https://mvlwb.com/registry/).

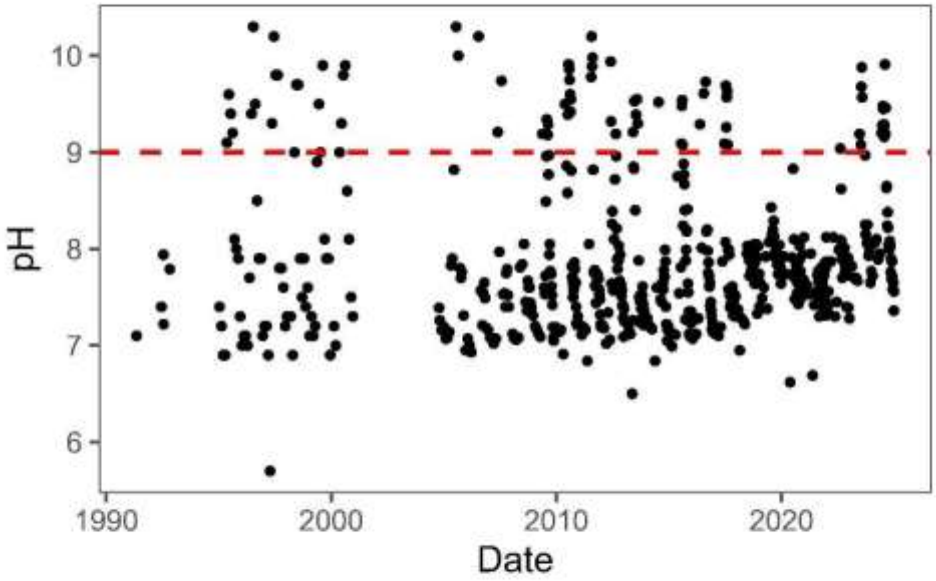

Fig. S13: pH at the outlet of Lake 'F3', downstream of Fiddler's Lake Sewage Lagoon. The red dashed line denotes the Water License's maximum grab samples effluent standard of 9. Data source: City of Yellowknife's Water License Reports (N1L4-0032, N1L3-0032, MV2009L3-0007, MV2021L3-0003) available online at Mackenzie Valley Land and Water Board Public Registry (https://mvlwb.com/registry/).

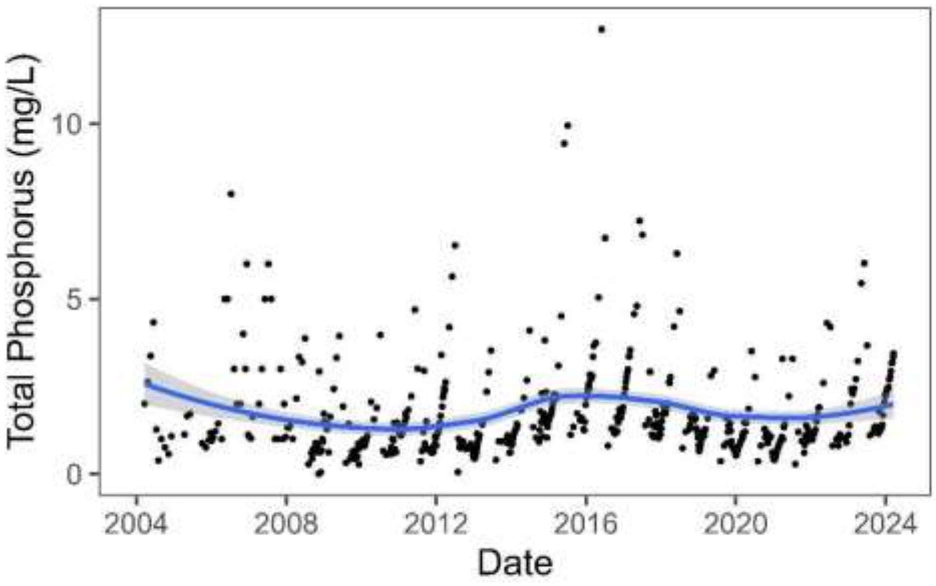

Fig. S14: Total phosphorus at the wetland' F1', downstream of Fiddler's Lake Sewage Lagoon. A LOESS model is coloured blue with a grey ribbon representing the standard error. Data source: City of Yellowknife's Water License Reports (N1L4-0032, N1L3-0032, MV2009L3-0007, MV2021L3-0003) available online at Mackenzie Valley Land and Water Board Public Registry (https://mvlwb.com/registry/).

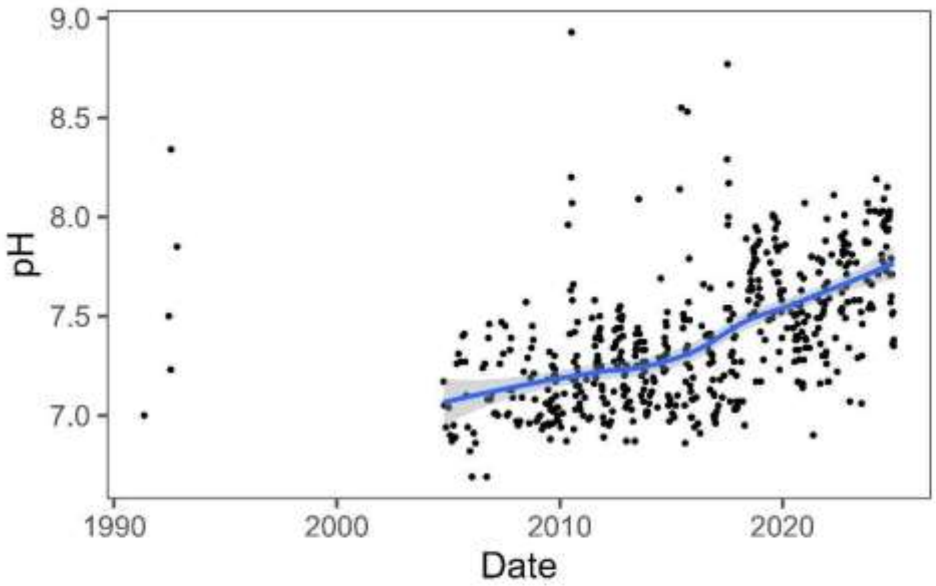

Fig. S15: pH of water at the wetland 'F1', downstream of the Fiddler's Lake Sewage Lagoon. A LOESS model is coloured blue with a grey ribbon representing the standard error. Data source: City of Yellowknife's Water License Reports (N1L4-0032, N1L3-0032, MV2009L3-0007, MV2021L3-0003) available online at Mackenzie Valley Land and Water Board Public Registry (https://mvlwb.com/registry/).

Fiddler's Lake Sewage Lagoon Timeline

Year	Description
**1981**	Fiddler's Lake Sewage Lagoon is constructed using a series of dams to merge small natural lakes to create a facultative lagoon. Following natural treatment, sewage effluent is discharged through a ~13.5 km series of lakes and wetlands before the final release into Great Slave Lake[1].
**1982**	Northwest Territory Water Board issues Water License (N1L4-0032)[1]. Mandatory total phosphorus effluent criteria at the sewage lagoon discharge point (F6) is set at 4 mg/L (single grab sample) and 2 mg/L (average of four samples)[1]. The sewage lagoon was reported decanted seasonally from May to October[2].
**1984**	Effluent criteria, at Fiddler's Lake Sewage Lagoon discharge point (F6), is exceeded for total phosphorus, pH, coliform count, and biological oxygen demand[3].
**1987**	Fiddler's Lake sewage lagoon capacity is increased by raising the dams (~2 m)[1]. Operating depth of the primary Fiddler's Lake Sewage Lagoon is ~4.5 m.
**1987**	Northwest Territory Water Board denies the City of Yellowknife's request to remove total phosphorus as an effluent criterion from Water License[1].
**1989**	Northwest Territory Water Board increases the phosphorus effluent criteria, from 4 mg/L to 5 mg/L (single grab sample) following a request from the City of Yellowknife[3, 4].
**1989**	Fiddler's Lake Sewage Lagoon is de-sludged for operational purposes, increasing total capacity[5].
**1989**	First observed unidentified minor blooms in Great Slave Lake, located within 1 km of the sewage outlet.
**1992**	Downstream Lake F3 (max depth 5 m) first identified as anoxic (<1 mg/L) in winter[6].
**1995**	Northwest Territory Water Board issues a new Water License (N1L3-0032)[1, 7]. Effluent compliance point moved downstream from the primary sewage lagoon (F6) to Lake F3. Total phosphorus is not included as a mandatory effluent criterion. The sewage lagoon was reported decanted seasonally from mid-September to mid-December.
**1995**	Downstream Lake F3 has pH spikes (>9) associated with suspected algal blooms in June, July, August[1].
**1998**	Fiddler's Lake Sewage Lagoon is adjusted by removing floating mat islands and cleaning the edge of the at the discharge point, increasing total capacity[1].
**2002**	Mackenzie Valley Land and Water Board issues new Water License (N1L3-0032)[8]. Total phosphorus continues to not be a mandatory effluent criterion but must be sampled at downstream lakes and wetlands (F3 and F11). Target effluent objective for total phosphorus is set at 2 mg/L (single sample) and 1 mg/L (average of four samples) to be achieved by 2008. The sewage lagoon was reported decanted seasonally from mid-September to mid-December.
**2010**	Mackenzie Valley Land and Water Board issues new Water License (MV2009L3-007)[9]. Total phosphorus is not a mandatory effluent criterion but must continue to be sampled at downstream lakes and wetlands (F3 and F11). Target effluent objective for total phosphorus remains at 2 mg/L (single sample) and 1 mg/L (average of four samples).
**2011**	In response to seasonal pH spikes (>9) additional field sampling was conducted along the series wetlands and lakes receiving sewage effluent[10]. Field sampling noted large quantities of submerged pondweed (*Elodea canadensis*), floating duckweed, suspended green microbial mats, and highly turbid brown/green water column. The potential for cyanobacteria blooms was discussed. No phytoplankton taxonomy samples were collected.

**2012**	Recorded concern of future increasing algal overgrowth and pH issues as a result of warmer summer temperatures, potentially related to climate change scenarios[11].
**2012**	Between 2001 and 2012, the sewage lagoon dam was frequently noted as having overflows, leaks, and small leaks to the series of downstream lakes and wetlands, at varying dates most often in May and June[12, 13].
**2017**	The approved start of decanting time of the lagoon is shifted earlier from previously typical times of mid-September to mid-July and continues until mid-December[5].
**2017**	A Great Slave Lake Nutrient Risk Assessment is issued by the City of Yellowknife's retained consultant, Associated Environmental[14]. The report noted that total phosphorus and dissolved phosphorus were elevated in the wetland lake system before the initiation of the seasonal decant from the lagoon. The report notes that long-term discharge from the Fiddler's Lake Sewage Lagoon could have saturated the wetland system beyond capacity.
**2020**	The Government of Northwest Territories (GNWT) in partnership with Environment and Climate retained Fleming College's Centre for Advancement of Water and Wastewater Technologies (CAWT) to collect and analyze winter water samples and sediment cores taken in the sewage wetland system and at the outfall in Great Slave Lake[15, 16]. The report found that phosphorus is likely migrating downstream to Great Slave Lake. The report noted that the total mass of phosphorus delivered annually as well as the capacity of the treatment system to retain phosphorus is unknown.
**2022**	Mackenzie Valley Land and Water Board issues new Water License (MV2021L3-0003)[17]. Total phosphorus is included as a mandatory effluent criterion set at 5.0 mg/L (single grab sample) and 3.5 mg/L (average of four samples) at Lake F3. Total phosphorus monitoring will also occur at downstream sites (F1 and F11)[17].
**2022**	A large unidentified bloom (7 km long) is observed in Great Slave Lake starting at the sewage outlet using Sentinel-2 satellite-based remote sensing.
**2023**	A Great Slave Lake Monitoring Program Design Plan is required under the Water License (MV2021L3-0003) to assess wastewater effluent on the receiving waters. At the time of publication, the proposed Great Slave Lake monitoring design plan did not include phytoplankton taxonomy or cyanotoxins[18].
**2024**	Seasonal pH spikes continue to be associated with unidentified algal blooms in the series of wetland lakes (F3)[19].

Table S2: Summarized timeline of the City of Yellowknife Fiddler's Lake Sewage Lagoon effluent and unidentified blooms.

References:

1. City of Yellowknife. *N1L3-0032 Water License Renewal Application Supplementary Information*. https://registry.mvlwb.ca/Documents/N1L3-0032/N1L3-0032%20-%20SupportingInfo.pdf (2001).
2. City of Yellowknife. *Reply to the Giant Mione Remediation containing the City of Yellowknife's 1982 N1L40032 Water License*. https://registry.mvlwb.ca/Documents/MV2007L8-0031/GMRP%20%20City%20of%20Yellowknife%20%20Reply%20to%20GMRP%20Claim%20for%20Compensation%20Response%20-%20Dec13-19.pdf (2019).

3. Northwest Territories Water Board. *N1L4-0032 Public Hearing Transcript on June 25, 1992.* https://registry.mvlwb.ca/Documents/N1L4-0032/N1L4-0032%20Public%20Hearing%20Transcript%20-%20June25_92.pdf (1992).
4. City of Yellowknife. *Written Submissions to the Northwest Territories Water Board in support of Water License L1L4-0032*. https://registry.mvlwb.ca/Documents/N1L4-0032/N1L4-0032%20City%20Submission%20for%20Public%20Hearing%20-%20June9_92.pdf (1992).
5. City of Yellowknife. *Fiddler's Lake Treatment System Management Plan V3*. https://registry.mvlwb.ca/Documents/MV2021L3-0003/City%20of%20YK%20-%20Type%20A%20Water%20Licence%20-%20Renewal%20Application%20-%20Fiddler's%20Lake%20Treatment%20System%20Management%20Plan%20-%20Feb5_21.pdf (2021).
6. Department of Fisheries and Oceans. *An evaluation of the fisheries associated with the Fiddler's Drainage System*. https://registry.mvlwb.ca/Documents/N1L4-0032/N1L4-0032%20Eval%20of%20Fisheries%20in%20Fiddlers%20-%20July19_93.pdf (1993).
7. City of Yellowknife. *Written Submissions to the Northwest Territories Water Board in support of Water License N1L4-0032*. https://registry.mvlwb.ca/Documents/N1L4-0032/N1L4-0032%20City%20Submission%20for%20Public%20Hearing%20-%20May10_94.pdf (1994).
8. Mackenzie River Land and Water Board. *Issuance of Type "A" Water License N1L3-0032.* https://registry.mvlwb.ca/Documents/N1L3-0032/N1L3-0032%20-%20License.pdf (2002).
9. Mackenzie River Land and Water Board. *Type "A" Water License MV2009L3-0007.* https://registry.mvlwb.ca/Documents/MV2009L3-0007/MV2009L3-0007%20-%20City%20of%20Yk%20-%20Water%20Licence%20with%20SNP%20updated%20-%20Sept1-11.pdf (2010).
10. Dillon Consulting Limited. *Fiddler's Lake Treatment System Studies and Management Plan: pH Compliance Point Assessment – Final Report. https://registry.mvlwb.ca/Documents/MV2009L3-0007/MV2009L3-0007%20-%20City%20of%20Yk%20-%20Sewage%20Lagoon%20pH%20Study%20-%20No%20Appendix%20D%20-%20May29-12.pdf (2012).*
11. Aboriginal and Northern Affairs Development Canada. *Comments for MV2009L3-0007- Fiddler's Lake Treatment System Studies and Management Plan- City of Yellowknife, July 2012.* https://registry.mvlwb.ca/Documents/MV2009L3-0007/MV2009L3-0007%20-%20City%20of%20YK%20-%20Sewage%20Lagoon%20pH%20Study%20-%20AANDC%20Comments%20-%20Jul10-12.pdf (2012).
12. City of Yellowknife. *Water Licence N1L3-0032: 2009 Annual Report.* https://registry.mvlwb.ca/Documents/N1L3-0032/N1L3-0032%20-%202009%20Annual%20Report%20-%20Mar31-10.pdf (2010).
13. City of Yellowknife. *Water Licence N1L3-0032: 2012 Annual Report. https://registry.mvlwb.ca/Documents/MV2009L3-0007/MV2009L3-0007%20-%20City%20of%20YK%20-%202012%20Annual%20Report%20-%20Apr2-13.pdf* (2013).
14. Associated Environmental. *City of Yellowknife Great Slave Lake Nutrient Risk Assessment Report.* https://registry.mvlwb.ca/Documents/MV2009L3-0007/MV2009L3-0007%20-%20City%20of%20YK%20-%20Fiddlers%20Lake%20Treatment%20System%20Plan%20V2%20-%20Jun28-18.pdf (2017).
15. Balch, G., Hill, R., Sheppard, M., Siembida-Lösch, B., Wootton, B. Assessing water quality parameters under ice in *Great Slave Lake at the outfall of the Fiddler's Lake treatment system - Report Prepared for the Government of Northwest Territories. https://registry.mvlwb.ca/Documents/MV2021L3-0003/City%20of%20YK%20-%20GNWT-ENR%20Application%20Review%20submission%20-%20CAWT%20Water%20Quality%20at%20Outfall%20of%20FLTS%20June%202020%20-%20Apr5-21.pdf (2020).*
16. Balch, G. Assessing phosphorus loadings in the Fiddler's Lake Treatment System, Yellowknife, NWT - Report Prepared for the Government of Northwest Territories. https://registry.mvlwb.ca/Documents/MV2021L3-0003/City%20of%20YK%20-%20GNWT-

ENR%20Application%20Review%20submission%20-%20CAWT%20FLTS%20P%20Loading%20Report%20-%20June%202020%20-%20Apr5-21.pdf (2020)

17. Mackenzie Valley Land and Water Board. *Issuance - Type A Water Licence MV2021L3-0003.* https://registry.mvlwb.ca/Documents/MV2021L3-0003/City%20of%20YK%20-%20Issuance%20-%20Type%20A%20Water%20Licence%20-%20Apr7_22.pdf (2022).

18. Associated Environmental. *Great Slave Lake Monitoring Program Design Plan V1.* https://registry.mvlwb.ca/Documents/MV2021L3-0003/City%20of%20YK%20-%20Great%20Slave%20Lake%20Monitoring%20Program%20Design%20Plan%20V1%20-%20Jan19_23.pdf (2023).

19. Government of the Northwest Territories – Department of Environment and Climate Change. *Correspondence re EQC exceedance at SNP station 0032-F3. https://registry.mvlwb.ca/Documents/MV2021L3-0003/City%20of%20YK%20-%20Correspondence%20re%20EQC%20exceedance%20at%20SNP%20station%200032-F3%20-%20Jun25_24.pdf (2024).*

Supplementary Information 4: Slave River sediment plume

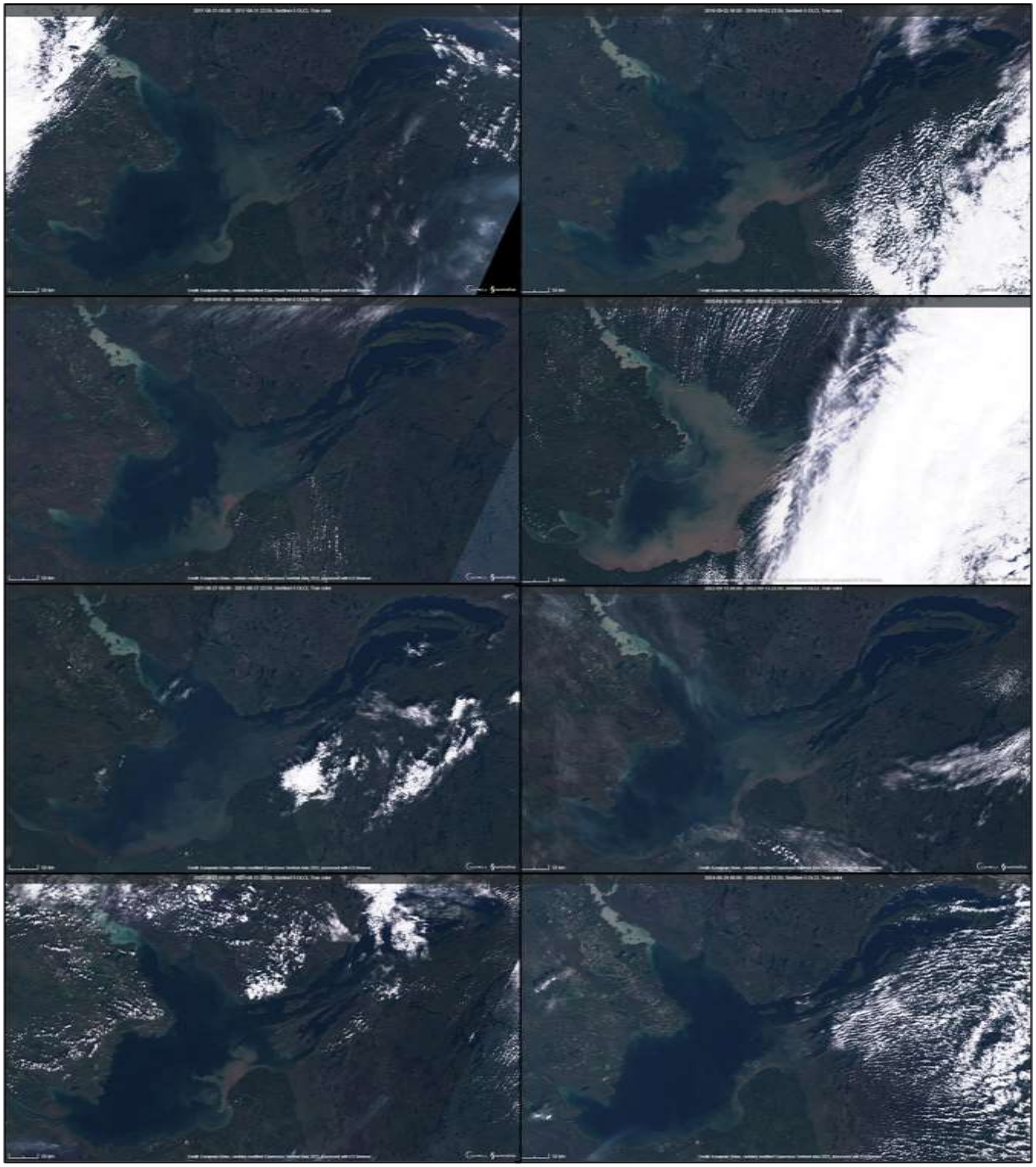

Fig. S16: Coverage of the Slave River sediment plume in Great Slave Lake visualized using Sentinel-3 Ocean and Land Colour Instrument in true colour on A) Aug. 31, 2017, B) Aug. 2, 2018, C) Aug. 9, 2019, D) Aug. 30, 2020, E) Aug. 27, 2021, F) Sep. 13, 2022, G) Aug. 23, 2023, H) Aug. 28, 2024. Credit: European Space Agency; contains modified Copernicus Sentinel data [2025] processed by Sentinel Hub.

Supplementary Information 5: Observed water quality changes across the NWT

Great Slave Lake sub-basin

In the sediment-laden Slave River, the headwaters of Great Slave Lake, Indigenous Knowledge holders began reporting increased unidentified green algae, at densities which clogged fishing nets, since 1993[1]. Along Great Slave Lake's rocky shorelines, an increase in attached periphyton has reportedly made boat and swimming access more difficult due to slippery conditions. Upstream of the Fiddler's Lake Sewage Lagoon outlet to Great Slave Lake, regulatory monitoring has repeatedly noted the observed overgrowth of floating duckweed and unidentified phytoplankton blooms, unfortunately no taxonomy samples were collected, or clear written bloom descriptions were included in reports[2]. Since 2019 residents of Dettah reported increased unidentified phytoplankton in Duck Lake, an upstream lake to Great Slave Lake, which receives discharge from that community's small sewage lagoon[3]. In Jackfish Lake, located within the City of Yellowknife limits, dense and recurrent whole-lake blooms of *Planktothrix* spp. were first observed in 2013[4]. Jackfish Lake has undergone a litany of cumulative impacts including eutrophication from urban runoff, mining contamination, warm water discharge from the city's diesel-powered hydro generating facility, and climate change— which cumulatively created a mesocosm of intensified change foreshadowing further increasing northern cyanobacteria blooms. Dense surface blooms, characteristic of cyanobacteria, have also been reported once in the Hay River[5] (Irene Graham, K'atl'oDeeche First Nation, *pers. comm.*). Elsewhere in the remote, and undeveloped Taiga Shield, a 2024 midsummer surface bloom, characteristic of cyanobacteria, was seen in an inland lake adjacent to the East Arm of Great Slave Lake (Jared Ellenor, Łutsël K'é resident, *pers. comm.*). In other inland lakes and ponds within the Taiga Shield near Yellowknife, we observed multiple nearshore cyanobacteria minor scums containing *Dolichospermum* spp. and *Aphanizomenon* spp. in early fall 2023 and 2024 (J. Cederwall *per. obs*.).

Southwest Northwest Territories in the Taiga Plains

The earliest written record of a suspected algal bloom in the Northwest Territories (NWT) was in Tathlina Lake in 1946. Tathlina Lake, is a large but shallow (mean depth 1.5 m) undeveloped eutrophic lake in the southern NWT, and an important walleye (*Sander vitreus*) fishery. Following a heatwave with calm winds, fisheries scientists observed a surface algal or cyanobacterial accumulation with enough density to discolour their boat's bow wave to light green; no taxonomy was collected[6]. Members of the Ka'a'gee Tu First Nation from the nearby community of Kakisa reported that minor algal blooms had occurred infrequently in Tathlina and Kakisa lakes and other lakes in the Taiga Plains but appear to be increasing over the last decade (Mike Low, Aboriginal Aquatic Resources and Ocean Management, *pers. comm.*). Elsewhere in the Dehcho region, Sambaa K'e First Nation first raised concerns of blooms, characteristic of cyanobacteria, and associated fish kills as a water quality issue in 1989[7] a concern that has persisted to the present day. In 2024, a harmful cyanobacteria bloom was confirmed in Sambaa K'e's drinking water reservoir (Jennifer Korosi, York University, *pers. comm.*). Elsewhere in the southwest NWT in 2024, algal blooms (not characteristic of cyanobacteria) occurred in several undeveloped, culturally significant lakes following severe wildfires in 2023 (Heidi Swanson, Wilfrid Laurier University, *pers. comm.*).

Northern Northwest Territories in the Taiga Shield

To date, there has been only one recorded cyanobacteria bloom in the NWT above the treeline which was associated with a (then) new diamond mine development at Snap Lake. During mandatory aquatic monitoring for Snap Lake diamond mine, elevated growth of *Aphanizomenon flos-aquae* was found in the summer of 2006, with a detectable concentration of microcystin-LR (0.9 µg/L max); which did not recur into future monitoring years[8].

Western Northwest Territories in the Mackenzie Mountains and Mackenzie Delta

In mountain streams west of the Mackenzie River and within the Mackenzie Delta, orange slime-like blooms of iron bacteria have been observed from iron-rich sediment inputs following climate-change-driven permafrost slumping[9].

References

1. Bill, L., Crozier, J. & Surrendi, D. *A Report of Wisdom Synthesized from the Traditional Knowledge Component Studies*. https://publications.gc.ca/collections/collection_2024/eccc/R71-49-4-12-eng.pdf (1996).
2. Dillon Consulting Limited. *A Report of Wisdom Synthes Fiddler's Lake Treatment System Studies and Management Plan: pH Compliance Point Assessment – Final Report.* https://registry.mvlwb.ca/Documents/MV2009L3-0007/MV2009L3-0007%20-%20City%20of%20Yk%20-%20Sewage%20Lagoon%20pH%20Study%20-%20No%20Appendix%20D%20-%20May29-12.pdf (2012)
3. Mackenzie Valley Land and Water Board. *MV2019L3-0007-Technical Workshop Yellowknives Dene First Nation Community of Dettah Type B Water Licence MV2019L3-0009 Renewal Technical Workshop Summary Notes*. https://registry.mvlwb.ca/Documents/MV2019L3-0009/MV2019L3-009%20-%20YKDFN%20Dettah%20-%20October%2028%202019%20Technical%20Workshop%20-%20Meeting%20Summary%20Notes%20-%20Nov13-19.pdf (2019).
4. Sivarajah, B., Simmatis, B., Favot, E. J., Palmer, M. J. & Smol, J. P. Eutrophication and climatic changes lead to unprecedented cyanobacterial blooms in a Canadian sub-Arctic landscape. *Harmful Algae* **105**, (2021).
5. Government of the Northwest Territories. *2019 NWT Water Stewardship 10th Annual Strategy Implementation Workshop Summary Report*. https://www.nwtwaterstewardship.ca/sites/water/files/resources/2019_wss_workshop_-_report_-_final_oct_2020.pdf (2020).
6. Kennedy, W. A. *A Report on Tathlina and Kakisa Lakes-1946*. https://waves-vagues.dfo-mpo.gc.ca/Library/59286.pdf (1962).
7. Lafontaine, C. & SKFN. *Sambaa K'e Municipal Environment Waste Management Practices and Monitoring Program (Draft)*. https://registry.mvlwb.ca/Documents/MV2012L3-0007/MV2012L3-0007%20-%20Sambaa%20Ke%20-%20Draft%20Municipal%20Environment%20Waste%20MGMT%20Practices%20and%20Monitoring%20Program%20-%20Jan21-14.pdf (2012).
8. Golder Associates. *Snap Lake Mine - Aquatic Effects Monitoring Program - 2007 Annual Report*. https://registry.mvlwb.ca/Documents/MV2001L2-0002/MV01L2-02%202007%20Annual%20AEMP%20Report%20Mar.01-08.pdf (2008).
9. Government of the Northwest Territories. *Iron Bacteria in Surface Water*. https://www.hss.gov.nt.ca/sites/hss/files/resources/iron-bacteria-fact-sheet-en.pdf (2024).
